# Extracting electrophysiological correlates of functional magnetic resonance imaging data using the canonical polyadic decomposition

**DOI:** 10.1002/hbm.25902

**Published:** 2022-05-14

**Authors:** Dylan Mann‐Krzisnik, Georgios D. Mitsis

**Affiliations:** ^1^ Graduate Program in Biological and Biomedical Engineering McGill University Montréal Quebec Canada; ^2^ Department of Bioengineering McGill University Montréal Quebec Canada

**Keywords:** computational modelling, electrophysiology, magnetic resonance imaging, motor‐imagery, neurovascular coupling, resting‐state, tensor decomposition

## Abstract

The relation between electrophysiology and BOLD‐fMRI requires further elucidation. One approach for studying this relation is to find time‐frequency features from electrophysiology that explain the variance of BOLD time‐series. Convolution of these features with a canonical hemodynamic response function (HRF) is often required to model neurovascular coupling mechanisms and thus account for time shifts between electrophysiological and BOLD‐fMRI data. We propose a framework for extracting the spatial distribution of these time‐frequency features while also estimating more flexible, region‐specific HRFs. The core component of this method is the decomposition of a tensor containing impulse response functions using the Canonical Polyadic Decomposition. The outputs of this decomposition provide insight into the relation between electrophysiology and BOLD‐fMRI and can be used to construct estimates of BOLD time‐series. We demonstrated the performance of this method on simulated data while also examining the effects of simulated measurement noise and physiological confounds. Afterwards, we validated our method on publicly available task‐based and resting‐state EEG‐fMRI data. We adjusted our method to accommodate the multisubject nature of these datasets, enabling the investigation of inter‐subject variability with regards to EEG‐to‐BOLD neurovascular coupling mechanisms. We thus also demonstrate how EEG features for modelling the BOLD signal differ across subjects.

## Graphical abstract

We show that EEG features of BOLD‐fMRI dynamics can be obtained using the canonical polyadic decomposition. Hemodynamic response functions are also estimated in the process, which allows one to model BOLD‐fMRI signals using EEG signals. We employ our method on simulated data, as well as publicly available task‐based and resting‐state EEG‐fMRI data.

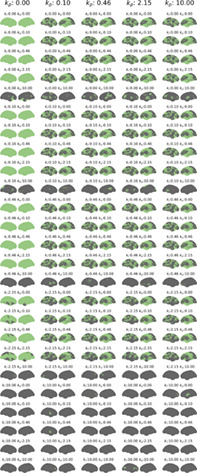

## 1 INTRODUCTION

Considerable effort has been dedicated to understanding the precise nature of the blood‐oxygen‐level dependent functional magnetic resonance imaging (BOLD‐fMRI) signal, due to its neurovascular and physiological origins (Hillman, 2014). One challenge is to remove confounds from BOLD‐fMRI data such as head motion (Power et al., 2014) and confounds related to systemic physiology, such as cardiac and respiratory activity (Kassinopoulos & Mitsis, 2019). In addition to denoising, other approaches for studying the BOLD signal include mathematically modelling the process of neurovascular coupling (Stephan et al., 2007), BOLD signal deconvolution (Sreenivasan et al., 2015) and combining BOLD‐fMRI with additional modalities (Uludağ & Roebroeck, 2014). From a multimodal perspective, simultaneous recordings of electroencephalography (EEG) and BOLD‐fMRI have gathered much attention and have provided important insight into electrophysiological correlates of the BOLD signal (Abreu et al., 2018).

One approach for bridging EEG and BOLD‐fMRI is to use EEG frequency‐band power signals as temporal predictors of the BOLD signal, in line with an asymmetrical fusion strategy (Abreu et al., 2018; Murta et al., 2015). As the EEG signal is considered as mostly instantaneous with regards to the underlying neural activity (despite some amount of blurring from brain tissue), whereas the BOLD signal exhibits some time lag, a temporal mismatch between EEG frequency‐bands and BOLD signals must be accounted for (Feige et al., 2017). The default strategy for accounting for this mismatch is to convolve frequency‐band amplitude signals with the canonical hemodynamic response function (HRF) (Goldman et al., 2002). However, the shape of the canonical HRF implies several assumptions about the dynamical relation between EEG frequency‐bands and the BOLD signal, overlooking the well‐known variability in HRF shape and thus the underlying neurovascular coupling mechanisms (Devonshire et al., 2012; Taylor et al., 2018).

We propose a method to identify the spatial and spectral electrophysiological correlates of the BOLD signal simultaneously with HRF estimation. This method is based on the canonical polyadic decomposition (CPD), a fundamental tensor decomposition which seeks a rank‐1 multilinear representation of tensor‐formatted data (Kolda & Bader, 2009). CPD is often considered analogous to a higher‐order variant of principal component analysis (PCA). Hence, CPD mainly differs from PCA in its ability to decompose higher‐order tensors—rather than just second‐order matrices—with the added feature of not necessitating orthogonality between the derived components. As a practical example of how it is employed, CPD may be applied to a third‐order tensor constructed by concatenating EEG time‐frequency maps for different channels (Cong et al., 2015). In this case, CPD returns a set of rank‐1 vectors describing the time‐courses, frequency bands and EEG channels which most explain the data. CPD has also been leveraged to extend group ICA analysis of BOLD‐fMRI data to a tensorial framework (Beckmann & Smith, 2005). Moreover, CPD and related tensor decompositions have been used for EEG‐fMRI data fusion (Acar et al., 2019; Karahan et al., 2015; Marecek et al., 2016; Van Eyndhoven et al., 2017).

More specifically, our method consists of constructing third‐order tensors which contain HRF estimates between frequency‐band dynamics and a given BOLD time‐course. CPD is applied to these HRF tensors to recover the main spatial and spectral distributions of HRF variability. This approach is similar to a higher‐order extension of multiway partial least‐squares as used for fusing EEG and BOLD‐fMRI data (Martínez‐Montes et al., 2004). Under certain conditions for the output noise, impulse response functions can generally be likened to cross‐correlations corrected for the input's serial autocorrelation (Westwick & Kearney, 2003). Performing CPD on a tensor comprising of such cross‐correlations amounts to computing eigenvectors which capture the main modes of the correlation structure embedded within the tensor. In other words, we perform CPD on a correlation tensor rather than a singular‐value decomposition or eigen decomposition on a correlation matrix. Formulated this way, our approach is also akin to the temporal kernel canonical correlation analysis proposed by (Biessmann et al., 2010) which was applied for fusing LFP recordings with BOLD data.

Here, we first simulated LFP‐fMRI data as a reference for assessing the ability of our method to estimate BOLD time‐series by deriving meaningful HRF estimates as well as their associated spatial and spectral distributions. We also evaluated the impact of cardiac and respiratory sources of physiological artefacts, which can undermine interpretability of results derived from analysing BOLD‐fMRI data. Then, we applied our method to three open‐source EEG‐fMRI datasets. Employing this method on either simulated LFP‐fMRI data or empirical EEG‐BOLD does not make a substantial difference in practice. The main idea remains largely unchanged: leveraging CPD to capture HRF variability across spatial distributions and frequencies.

Moreover, in light of the multisubject nature of the empirical open‐source datasets, we extend HRF tensors to fourth order to cover an additional subject‐related dimension. Hence, CPD applied to fourth order HRF tensors returns a distribution of subject weightings in addition to the spatial, spectral distributions and HRF estimates. This allows us to use the proposed framework for studying subject‐specific neurovascular coupling mechanisms, while simultaneously accounting for broadband electrophysiological activity combined with flexibility in the HRF dynamics with respect to both electrophysiological frequency and spatial location.

## 2 METHODS

**FIGURE 1:**
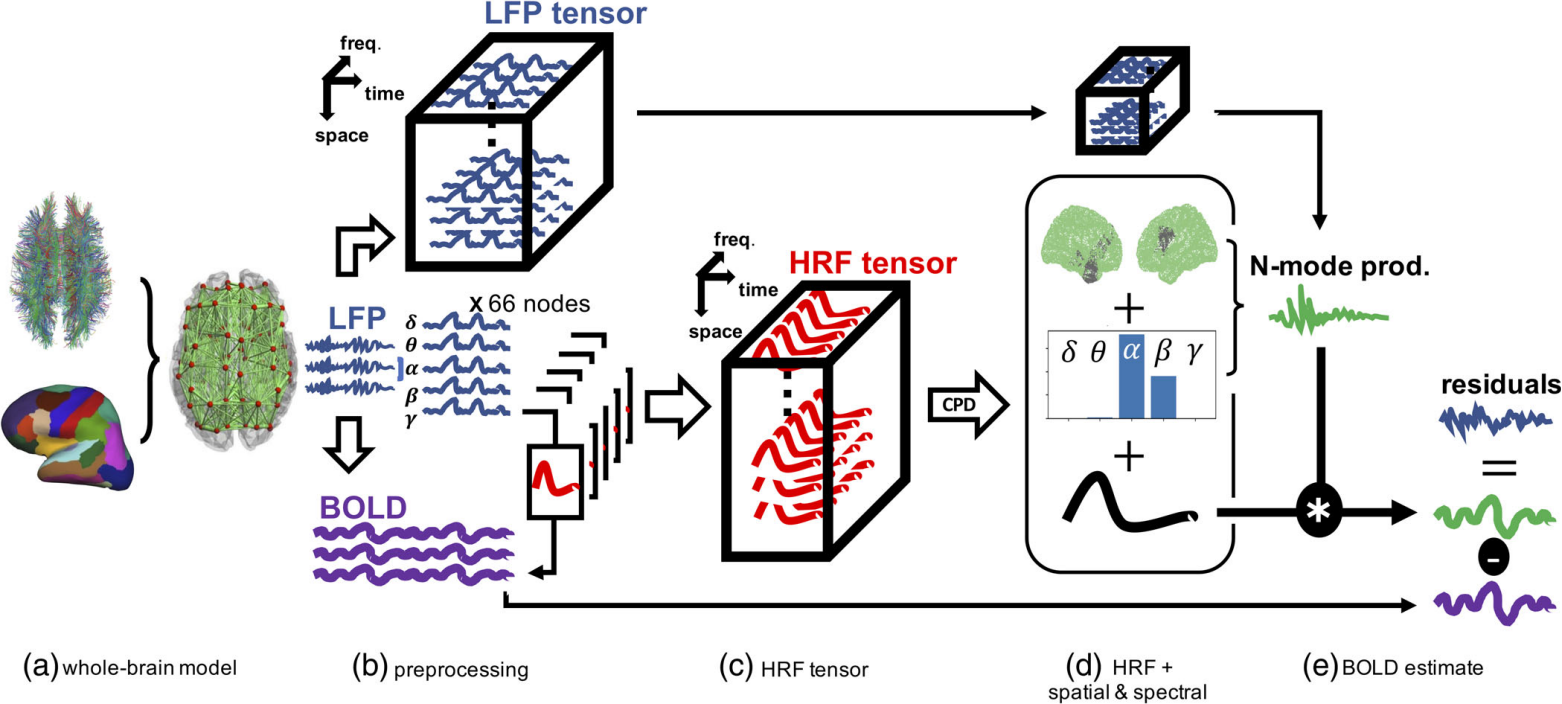
General workflow for simulated data. (a) A whole‐brain model (WBM) was constructed by combining MRI tractography and Desikan–Killiany cortical parcellation. The local dynamics of the nodes of the WBM were simulated using modified Stuart–Landau oscillators, giving rise to simulated local field potentials (LFP). (b) BOLD data were then generated from the LFPs. LFP data were bandpass filtered and Hilbert‐transformed to obtain amplitude signals which were stored in an LFP tensor for later use. Spatial–spectral response functions were estimated between each frequency‐band amplitude signal and each BOLD signal. (c) Estimated spatial–spectral response functions associated with a given BOLD signal were stored within a third‐order HRF tensor, organized along dimensions of space (node index) and frequency band. (d) The HRF tensor underwent a CPD to extract the dominant spatial and spectral distributions of HRF variability, as well as the dominant HRF waveform. (e) The N‐mode product was applied to the LFP tensor using the CPD‐derived spatial and spectral distributions, enacting a weighted averaging across spatial and spectral dimensions using these distributions as weights. We term the resulting signal the compound LFP signal. The latter was convolved with the CPD‐derived HRF to estimate its contribution to BOLD signal variability. The BOLD signal estimate and real BOLD signal were subtracted to return residuals, later used for bootstrapping. Panel 1a adapted from figure 2 of Cabral et al. (2014) with permission from the corresponding author

We propose a novel methodological framework to simultaneously obtain the spatial and spectral electrophysiological correlates of BOLD‐fMRI data and perform HRF estimation. When applied to simulated data as proof‐of‐concept, the main constituents of the proposed framework are the whole‐brain modelling of electrophysiology and BOLD‐fMRI data, pre‐processing of multivariate electrophysiological data, HRF estimation based on CPD of spatial–spectral response functions and phase‐randomisation of BOLD data for performing statistical inference. The different steps of the framework are illustrated in Figure 1.

**FIGURE 2:**
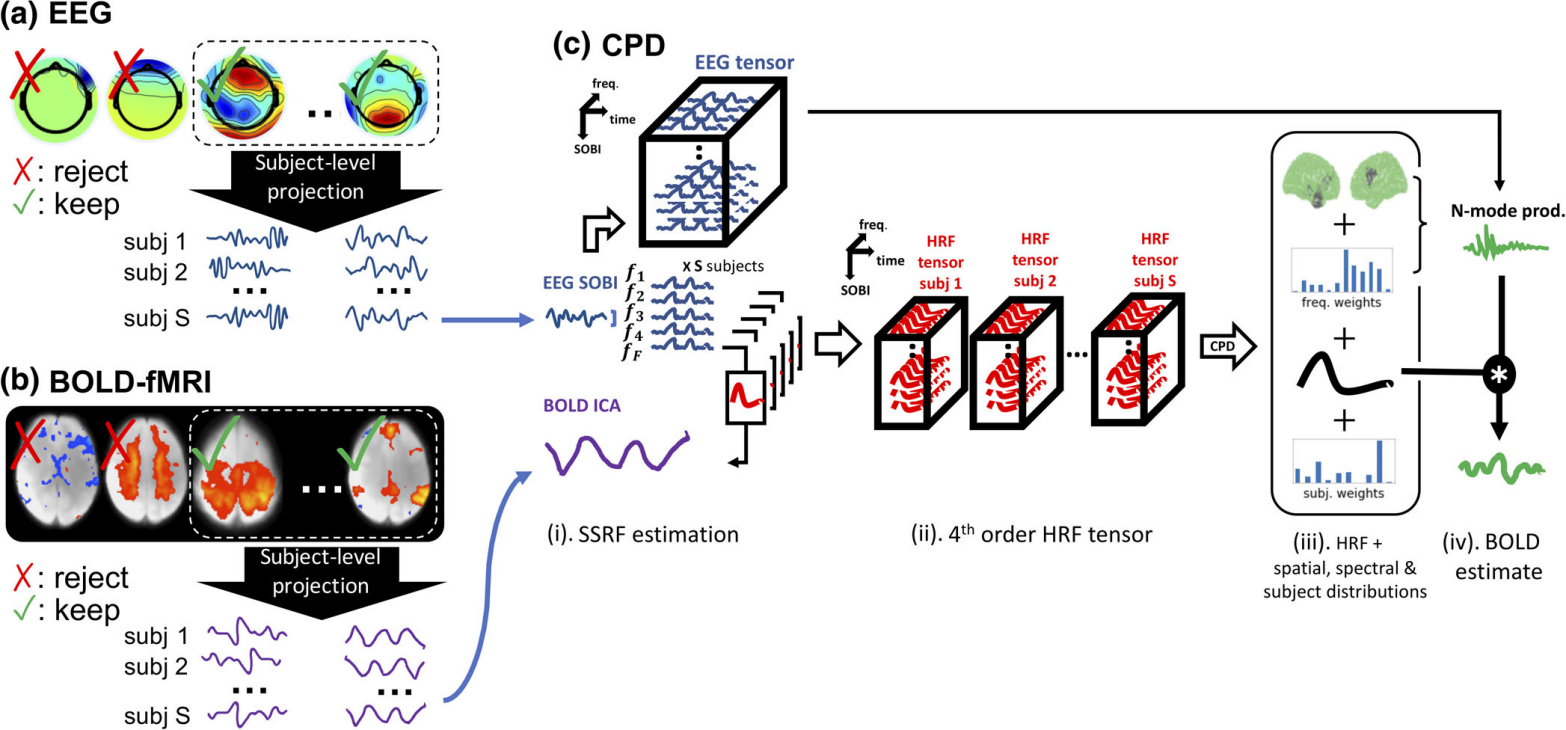
General workflow for empirical data. (a) Concatenated EEG data decomposed using the second‐order blind identification algorithm (SOBI). The retained SOBI components are projected onto individual subjects to obtain subject‐specific time‐series for each component. (b) Concatenated BOLD‐fMRI data decomposed using group ICA. The retained ICA components are projected onto individual subjects using spatial regression (i.e. the first step of dual regression) to obtain subject‐specific time‐series for each component. (c) Construction of fourth‐order HRF tensor and its decomposition using canonical polyadic decomposition. (c‐i) Time‐frequency decomposition of individual EEG SOBI time‐courses (a) is performed to extract oscillation amplitude signals within each pre‐specified frequency bin. A spatial/spectral response function (SSRF) is estimated between each amplitude signal and the BOLD time‐series of interest. Amplitude signals are stored within an EEG tensor (one tensor per subject). (c‐ii) For each BOLD ICA time‐series, SSRF estimates are stored into a fourth order HRF tensor with dimensions of time, frequency, SOBI component (also referred as space) and subject. (c‐iii) The HRF tensor is decomposed using CPD to obtain weighted distributions of SOBI components, frequencies and subjects as well as an HRF. (c‐iv) Distributions of SOBI components and frequencies are used to perform N‐mode products on the EEG tensor. This provides a compound EEG signal which is convolved with the estimated HRF to provide an estimate of the BOLD time‐series of interest, yielding a BOLD signal estimate for each subject

Moreover, an overview of our analysis when applied to empirical data is shown in Figure 2. Figure 2a,b shows additional steps where EEG and BOLD‐fMRI group decompositions are depicted. The results of these group decompositions are then forwarded to the rest of the analysis as shown in Figure 2c. The subfigures of Figure 2c closely match those of Figure 1 with the addition of a subject dimension.

### 2.1 Simulated data

#### 2.1.1 Whole‐brain modelling

To simulate realistic whole‐brain measurements of electrophysiological and neuroimaging data, an approach based on MR diffusion imaging tractography and mean‐field modelling was adopted (Deco, Kringelbach, et al., 2017; Sanchez‐Rodriguez et al., 2018; Schirner et al., 2018). Mesoscopic dynamics of individual brain regions were simulated using mean‐field models (MFM), where the local dynamics of individual brain regions interact through coupling mechanisms. The interaction strength between different brain regions was determined by a structural connectivity matrix based on MR diffusion tractography. This approach leverages our understanding of the brain as a dynamic, modular and integrative organ whose network‐like activity gives rise to processing of sensory information, motor control, cognition and other cerebral processes (Deco et al., 2015).

For data simulation, we used *The Virtual Brain* (TVB), a neuroinformatics platform for full brain network simulations using biologically realistic connectivity (Sanz‐Leon et al., 2013). Most data required to run the simulations are provided by TVB within a demonstration dataset. The TVB demonstration dataset includes coordinates for cortical parcellation nodes and a structural connectivity matrix based on fiber tractography derived from MR diffusion imaging.

The sample structural connectivity matrix quantifies interaction strengths between 66 cortical brain regions—or *nodes* in network‐science terminology—determined by the Desikan–Killiany cortical parcellation (Desikan et al., 2006). To enact local node‐level dynamics, we used Stuart–Landau oscillators (SLO) as MFM (Moon et al., 2015). The SLO serves as a canonical model for dynamical systems which undergo a supercritical Hopf bifurcation upon changing a specific parameter of the model describing the given dynamical system, a so‐called bifurcation parameter (Kuznetsov, 2004). Simulating stochastic SLOs close to the bifurcation can recreate rich neurodynamics using a limited number of model parameters.

The differential equations describing the behaviour of coupled stochastic SLOs in Cartesian coordinates are shown in Equations (1) and (2). In Cartesian form, two variables *x* and *y* interact via nonlinear equations.

(1) $$\frac{dx_{j}}{\mathrm{dt}}=\left[\alpha_{j}-x_{j}^{2}-y_{j}^{2}\right]x_{j}-\omega_{j}y_{j}+G\sum_{i}C_{\mathrm{ij}}x_{i}+\beta \eta_{j}$$

(2) $$\frac{dy_{j}}{\mathrm{dt}}=\left[\alpha_{j}-x_{j}^{2}-y_{j}^{2}\right]y_{j}+\omega_{j}x_{j}+G\sum_{i}C_{\mathrm{ij}}y_{i}+\beta \eta_{j}$$

The first two terms of each equation enact the local dynamics of a single SLO for a given node j. The local dynamics are dictated by the bifurcation parameter α and natural angular frequency ω. The bifurcation parameter controls the amplitude r of the oscillations, which is equal to $r=\sqrt{\alpha }$ for α>0 and r=0 for α≤0.

The third terms of (1) and (2) correspond to the coupling of the oscillator from node *j* with oscillators of other nodes 𝑖 ≠𝑗. The pairwise linear coupling is scaled by entries of the diffusion MR‐based structural connectivity matrix 𝐶_𝑖𝑗_. The sum of linear couplings is finally scaled by a global coupling parameter G, which tunes the contribution of coupled interactions to the overall dynamics of node *j*. We found a value of G=0.3 to provide a good balance between SLO‐driven local dynamics and collective coupled behaviour, resulting in realistic neurodynamics. Readers may refer to Deco, Cabral, et al. (2017) and Deco, Kringelbach, et al. (2017) for discussions regarding the global coupling parameter. The last term $\eta_{j}$ is an additive white Gaussian noise process (AWGN), which drives the oscillator through different dynamical regimes if the parameter α is close to its bifurcation value, that is, α≈0. We propose a variant of SLO to model richer oscillatory amplitude dynamics. For each node of our parcellation, two subpopulations of SLOs with different angular frequencies interact. The interaction is characterised by a *slow* SLO subpopulation driving the behaviour of a *fast* SLO subpopulation. Specifically, the bifurcation parameter α of the fast subpopulation is directly coupled to the amplitude of the slow subpopulation. In this way, the slow subpopulation serves as a modulator signal for oscillations of the fast subpopulation since αdirectly tunes oscillation amplitude r (i.e. $r\left(t\right)=\sqrt{\alpha \left(t\right)}$ for $\alpha \left(t\right)>0$ and $r\left(t\right)=0$ for $\alpha \left(t\right)\leq 0$).

Equations (3) and (4) formulate this concept of a fast SLO (Equation (3)) being modulated by a slow SLO (Equation (4)). The slow subpopulation variables $u_{j}$ and $v_{j}$, scaled by the fixed parameter $\alpha_{j}^{f}$, serve as a time‐varying bifurcation parameter which directly modulates the amplitude dynamics of the fast subpopulation variables $x_{j}$ and $y_{j}$. An example time‐series of a slow subpopulation is shown in Figure S1a whereas that of a fast subpopulation is shown in Figure S1b. The primary distinction between fast and slow SLOs relates to their respective natural frequency parameters. Moreover, since the bifurcation parameter α of the fast SLOs is driven by the amplitude of slow SLOs, it becomes time‐varying rather than fixed throughout a given simulation, that is, $\alpha =\alpha \left(t\right)$ in Equation (3).

Fast SLOs (Equation (3)) oscillate at time scales corresponding to common electrophysiological frequency bands (e.g. $\omega_{j}^{f}$= 1–100 Hz), whereas the natural frequency of slow SLOs (Equation (4)) is orders of magnitude smaller (e.g. $\omega_{j}^{s}$ = 0.01–0.1 Hz), on the scale of BOLD signal fluctuations. To introduce variability in peak oscillation frequency across nodes within standard frequency ranges, $\omega_{j}^{f}$ for nodes belonging to the right hemisphere were set to 2 Hz whereas $\omega_{j}^{f}$ for nodes belonging to the left hemisphere were set to 10 Hz. The $\omega_{j}^{s}$ parameter was set to 0.08 Hz for all nodes, corresponding to an oscillation period of 12.5 s and thus roughly mimicking BOLD oscillations observed during resting‐state.

(3) $$\text{Fast}\;\mathrm{SLO}\left\{\begin{array}{l} \frac{{\mathrm{dx}}_{j}}{\mathrm{dt}}=\left[\alpha_{j}^{f}u_{j}-x_{j}^{2}-y_{j}^{2}\right]x_{j}-\omega_{j}^{f}y_{j}+{\beta \eta }_{j}\\ \frac{{\mathrm{dy}}_{j}}{\mathrm{dt}}=\left[\alpha_{j}^{f}v_{j}-x_{j}^{2}-y_{j}^{2}\right]y_{j}+\omega_{j}^{f}x_{j}+{\beta \eta }_{j} \end{array}\right.$$

(4) $$\text{Slow}\;\mathrm{SLO}\left\{\begin{array}{l} \frac{{\mathrm{du}}_{j}}{\mathrm{dt}}=\left[\alpha_{j}^{s}-u_{j}^{2}-v_{j}^{2}\right]u_{j}-\omega_{j}^{s}v_{j}+G\sum_{i}C_{\mathrm{ij}}u_{j}+{\beta \eta }_{j}\\ \frac{{\mathrm{dv}}_{j}}{\mathrm{dt}}=\left[\alpha_{j}^{s}-u_{j}^{2}-v_{j}^{2}\right]v_{j}+\omega_{j}^{s}u_{j}+G\sum_{i}C_{\mathrm{ij}}v_{j}+{\beta \eta }_{j} \end{array}\right.$$

The coupling across nodes is another noteworthy aspect of the current implementation of modified SLOs, where only slow subpopulations may interact across nodes. This inter‐node coupling was implemented in light of our focus on amplitude dynamics and evidence by which amplitude functional connectivity patterns derived from electrophysiology can reproduce functional connectivity patterns when using BOLD‐fMRI (Brookes et al., 2011). Hence, amplitude dynamics are of primary interest for relating electrophysiology to BOLD dynamics, whereas processing the oscillatory signals as done with empirical data is a useful exercise to assess the effects of common pre‐processing steps on our analysis. The implications of some pre‐processing steps on the analysis, namely band‐pass filtering, will be discussed later on.

Moreover, the balloon model (Buxton & Frank, 1997; Friston et al., 2000) was used to simulate BOLD data using simulated LFPs as input time‐series. As a proxy for oscillatory amplitude dynamics, LFP signals were squared before serving as inputs to the balloon model. The balloon model accounts for the increase of regional cortical blood flow in response to neuronal activity, the ensuing changes in haemodynamic state variables and the measurable BOLD signal which arises as a result of these mechanisms. Figure S1c shows an example of simulated BOLD time‐series. We acknowledge the existence of more complex and realistic models of neurovascular coupling (Huneau et al., 2015). However, the objective of using the balloon model in the present study was to investigate the effects of regional HRF variability by varying some of its physiologically interpretable parameters (notably, the autoregulation time‐constant as discussed in the next paragraph). Furthermore, the balloon model has been extensively used by the neuroimaging community and it is routinely fitted to empirical data when using Dynamic Causal Modelling (DCM; Friston et al., 2003), with some degree of consensus that, following appropriate estimation of its parameters, it can sufficiently fit experimental BOLD data. Finally, the balloon model can create neurovascular coupling dynamics which have been studied using Volterra kernels (Friston et al., 2000), with the results suggesting that a linear time‐invariant (LTI) system is a good linear approximation (Friston, Fletcher, et al., 1998). In our simulations, it was found that a linear HRF was able to capture most of the balloon model's output, suggesting that an LTI system (e.g. an HRF) is sufficient for the input signal range used in the present study. To support this statement, we injected AWGN as input into the balloon model and used the output to estimate an HRF. This process was repeated multiple times with different parametrisations of the balloon model. For each simulation, convolving the input AWGN with its associated HRF and correlating the resulting signal with the balloon model output yielded a median correlation coefficient of 0.9.

The dynamics enacted by the balloon model may be manipulated by tuning the model parameters. Hence, to impose regional cerebrovascular variability, the autoregulation time‐constant of the balloon model was sampled from a log‐normal distribution for all nodes of the cortical parcellation. This log‐normal distribution had a mean value of 2.46 and standard deviation 0.212 following Table 2 from (Khalidov et al., 2011). This sampling procedure resulted in a node‐specific HRF for each cortical node.

Physiological signals were also modelled to evaluate the impacts of cardiac and respiratory related systemic fluctuations on HRF estimation and spatial–spectral feature extraction (Figure S2). It is known that these physiological processes can induce systemic fluctuations in hemodynamics (Chen et al., 2020; Kassinopoulos & Mitsis, 2022; Xifra‐Porxas et al., 2020); these processes are termed systemic low‐frequency oscillations (SLFOs^1^; Tong et al., 2016). Standard SLOs were used to simulate time‐varying respiration waveforms (RW, Equations (1) and (2)). A nonlinear oscillator taken from (Rundo et al., 2018) was used to simulate cardiac pulses as seen when performing photoplethysmography (PPG, Equations ((5), (6), (7))), where $x_{2}$ of Equation (6) was taken to be the PPG. Importantly, the integration time‐constant of the oscillators for PPG modelling was directly modulated by the simulated RW. This coupling between respiratory and cardiac activity serves to simulate respiratory sinus arrhythmia, by which inhalation upregulates heart‐rate whereas exhalation downregulates heart‐rate in healthy adults (Rassler et al., 2018).

PPG (Figure S2a) and RWs (Figure S2b) were simulated using a stochastic Stratonovich–Heun algorithm (Burrage et al., 2004) implemented in the *sdeint* Python package. Model parameters were set to $\mu =0.5,p_{1}=-0.3,p_{2}=0.3,b=1$ as per (Rundo et al., 2018). Otherwise, we set a=7,c=14 as we have found these values to produce the sought‐for coupling between RW and PPG. Furthermore, k=1.5 to recreate the systolic and diastolic peaks of the PPG waveforms.

(5) $$\frac{dx_{1}}{\mathrm{dt}}=\frac{1}{\tau \left(t\right)}\left(-x_{1}+\left(1+\mu \right)y_{1}-by_{2}+p_{1}\right)$$

(6) $$\frac{dx_{2}}{\mathrm{dt}}=\frac{1}{\tau \left(t\right)}\left(-x_{2}+\left(1+\mu \right)y_{2}+by_{1}+p_{2}\right)$$

(7) $$\frac{1}{\tau \left(t\right)}=a\frac{d}{\mathrm{dt}}\mathrm{RW}\left(t\right)+c$$

$$y_{1}=\tanh\left(kx_{1}\right)$$

$$y_{2}=\tanh\left(kx_{2}\right)$$

In short, LFP data were simulated for 66 nodes using modified SLOs, coupled via whole‐brain structural connectivity. LFPs were used as input signals to the balloon model to simulate BOLD data. Physiological signals were also simulated by modelling respiratory waveforms and PPG.

#### 2.1.2 Pre‐processing

Initially, LFP signals were subjected to time‐frequency transformations after passband filtering the individual signals within standard frequency bands and obtaining the Hilbert transform of the resulting narrowband signals. The following standard frequency bands were used: delta (1–4 Hz), theta (4–8 Hz), alpha (8–12 Hz), beta (12–32 Hz) and gamma (32–50 Hz). Using these frequency bands allowed us to relate our results with previous relevant studies more directly, and assess how processing data (filtering, time‐frequency transforms, decimation, etc.) affects common analyses. Nonetheless, our framework permits users to define alternative frequency bins.

The Hilbert‐transformed narrowband signals were truncated to remove initial transient behaviour resulting from numerical integration of SLOs and the balloon model. Linear trends were also removed. Subsequently, the data were low‐pass filtered to avoid aliasing (decimation) and the resulting low‐pass signals were down‐sampled to 4 Hz to reduce computational load. The BOLD signals were also truncated and decimated in the same fashion.

To study the effects of SLFOs on our analysis, simulated cardiac pulses (Figure S2a) and respiratory waveforms (Figure S2b) were transformed into signals related to SLFOs (Kassinopoulos & Mitsis, 2019). Cardiac pulses were transformed into beat‐to‐beat heart‐rate (HR) signals by calculating the reciprocal of the time interval between successive cardiac peaks (Figure S2c). The HR signal was linearly interpolated to match the sampling rate of LFP and BOLD data. The temporal derivative of respiratory waveforms was squared, giving rise to a feature termed respiratory flow (RF, Figure S2d). HR was convolved with a cardiac response function (CRF, Figure S2e) and RF convolved with a respiratory response function (RRF, Figure S2f) to create the SLFOs. HR and RF SLFOs were summed together to output a single SLFO signal (Figure S2g). This SLFO signal was subsequently truncated, detrended and decimated before being added as physiological confound. The standard CRF and RRF used herein are described respectively in Chang et al. (2009) and Birn et al. (2008) although readers may also refer to (Kassinopoulos & Mitsis, 2019) for discussion regarding physiological response functions.

With evidence suggesting that SLFOs do not affect the BOLD signal uniformly across brain regions (Kassinopoulos & Mitsis, 2019; Tong et al., 2016), we derived scaling coefficients for each node which were subsequently used to adjust the amplitude of the SLFO signal for individual nodes. Specifically, the amplitude of the SLFO signal was increased for brain regions for which the BOLD signal is more affected by SLFOs. To obtain these coefficients, a Desikan–Killiany overlay was aligned with a map of coefficients quantifying the association between a given voxel's BOLD signal and SLFOs, as reported in (Kassinopoulos & Mitsis, 2019). Separate maps were used for the cardiac related SLFOs and respiratory SLFOs. These maps were first down‐sampled to a 1 mm resolution using linear interpolation and smoothing. For both cardiac and respiratory SLFOs, the average coefficient value was computed for each of the 66 parcels of the Desikan–Killiany parcellation. By accounting for the impact of regional heterogeneity of SLFOs on the BOLD signal, we aimed to generate more realistic simulation data and examine the effect of this heterogeneity on HRF estimation as well as extraction of spatial–spectral distributions. The Desikan–Killiany overlay in MNI 152 2 mm space was taken from the GitHub repository of the *AtlasReader* package.^2^

#### 2.1.3 HRF estimation and derivation of spatial–spectral distributions

The HRF estimation scheme relies on the Canonical Polyadic Decomposition (CPD), a tensor decomposition technique which extracts the low‐rank structure of multivariate data (Kolda & Bader, 2009). In machine learning, a tensor generalizes the concepts of vectors (1D) and matrices (2D) to include higher orders, that is, greater than 2D.

The CPD is often compared to principal components analysis for higher‐order data structures. CPD models a given tensor into a sum of *N* components, where each component is a low‐rank tensor resulting from the outer product of *C* modes. Equation (8) shows the CPD model of tensor X with modes a,b and c. Figure S3 shows a graphical illustration of the CPD model.

(8) $$X=\sum_{i=1}^{N}a_{i}\circ b_{i}\circ c_{i}$$

For simplicity, the number of components *N* was set to 1 when evaluating our method on simulated data. However, similarly to outputting multiple components during PCA, *N > 1* can generally be used to derive multiple CPD components as will be seen when using empirical data.

The HRF tensor (Figure 1c) is one of the mathematical quantities of major importance for the proposed framework. The HRF tensor captures the variability in neurovascular coupling mechanisms relating electrophysiology to BOLD. To construct an HRF tensor, a spatial–spectral response function (SSRF) was estimated between the LFP signal associated with each space and frequency pair and the BOLD signal of a given node. In other words, the five frequency bands and 66 parcellation nodes resulted in 330 different amplitude signals obtained from performing the Hilbert transform. An impulse response function (i.e. SSRF) was estimated between each of these 330 amplitude LFP signals and the BOLD signal under consideration, resulting in 330 SSRFs associated with a single BOLD signal. These SSRFs were stored within a third‐order HRF tensor and organised as a function of parcellation node and frequency band. Readers should refer to Figure 1c for a visual depiction of how HRF tensors are constructed.

To estimate individual SSRFs, we adopted the basis functions expansion technique (Marmarelis, 2004) and used spherical Laguerre basis functions (Leistedt & McEwen, 2012; Prokopiou, Xifra‐Porxas, et al., 2022). The damped oscillatory shape of Laguerre functions makes them suitable for modelling physiological systems with low‐pass behaviour and finite memory. The spherical variant of Laguerre functions also has the desirable property of beginning at zero amplitude, rendering them useful for estimating HRFs using a limited number of basis functions. Once the number of spherical Laguerre functions is defined, only a single parameter is needed to generate these functions, namely, the decay rate α. The first three spherical Laguerre basis functions are shown in Figure S4 for different decay parameter values.

Before running the phase‐randomisation routine (explained in the next section), we performed a parameter sweep to select the optimal decay rate for every node. We searched the parameter space $\alpha \in \left[\;0.40,3.00\;\right]$ with steps of 0.05. The decay rate maximising the correlation between the BOLD signal and BOLD signal estimate was chosen on a node‐by‐node basis for further analysis. The number of basis functions during analysis was set to 3, as it was empirically observed that this number provided a good trade‐off between HRF complexity and robustness during estimation. Also, for estimating SSRFs, the LFP and BOLD signals were z‐score normalised to account for differences in variance. The SSRF and HRF length was set to 32 seconds.

SSRF estimation, calculation of correlation coefficients, soft‐thresholding and scaling of SSRFs resulted in 330 SSRFs (66 LFP nodes × 5 frequency bands) which were stored in an HRF tensor. The dimensions of this tensor are equal to the number of nodes, number of frequency bands and HRF length (66 nodes × 5 bands × 32 s). Thereafter, the HRF tensor was denoised and sparsified using a rescaling procedure based on soft‐thresholding as explained in Supporting Information (Supplementary Methods 1—Rescaling the HRF tensor using soft‐thresholding). CPD was then applied to this rescaled HRF tensor to obtain the dominant HRF shape as well as spatial and spectral distributions associated with this HRF for the BOLD signal under consideration (Figure 1d). The resulting spatial and spectral distributions allowed us to obtain a weighted linear combination of the LFP signals, giving rise to what we call the compound LFP signal. The weighted linear combination was performed by applying the N‐mode product (Kolda & Bader, 2009) to the LFP tensor across both space and frequency dimensions (Figure 1d,e). The compound signal convolved with the dominant HRF was then correlated to the BOLD signal of interest, as a measure of goodness‐of‐fit. CPD and the N‐mode product were performed using the *Tensorly* 3 package (Kossaifi et al., 2019).

We tested the robustness of our proposed HRF estimation scheme in the presence of input and output measurement noise as well as SLFOs. Input and output measurement noise was first simulated using AWGN processes. The relative impact of each of these three noise signals was controlled by amplifying them by different amounts. The scaling coefficients associated to each of the three noise signals were stored in an array of tuples, each tuple comprising of a unique combination of scaling values. For each tuple, the scaling coefficients were all individually set to one of the five following values: 0, 0.1, 0.46, 2.15 and 10—the latter four values were derived from a log‐spaced sequence between 0.1 and 10. The list of tuples was created from all combinations of these five scaling coefficients applied to the three confound signals (i.e. $5^{3}=125$ combinations). We thus iterated through this array of tuples to scale the input and output noise by different values. See Figure S6 for an illustration of this process of injecting different source of confounds. It should be noted that this process was enacted prior to re‐weighting the HRF tensor. Also, it is worth remembering that the SLFO was originally scaled node‐wise by different values according to maps of coefficients quantifying the association between BOLD signal and SLFO taken from (Kassinopoulos & Mitsis, 2019). On the other hand, the scaling coefficient $k_{p}$ for the SLFO shown in Figure S6 scales SLFOs uniformly across nodes, controlling the global influence of SLFO. Thus, the final node‐wise scaling of SLFO results from the product of the original node‐specific scaling values and the uniform scaling coefficient $k_{p}$.Moreover, whereas SLFOs and output measurement noise was directly added to the BOLD data after scaling, the AWGN input measurement noise underwent some processing steps explained in the Supporting Information (Supplementary Methods 2—Injecting input noise). These processed input measurement noise signals were scaled and added to the LFP/EEG during the HRF‐estimation part of the analysis.

Our handling of input and output measurement noise varied according to the expected effects of these noise types on the LFP and BOLD signals. When analyzing BOLD data, some thermal noise is expected to remain in the data in the form of a white‐noise process (although high‐pass filtering of BOLD data in standard pre‐processing pipelines would remove the very low frequency trends of this thermal noise). On the other hand, time‐frequency analysis of LFP data often involves a nonlinear transformation (e.g. amplitude of the complex signal arising from the Hilbert transform), thereby impacting the thermal noise in non‐trivial ways. Hence, whereas the output measurement noise was modelled simply by an AWGN process, the input measurement noise was modelled by accounting for the way in which the Hilbert transform alters an AWGN process (Supporting Information, Supplementary Methods 2—Injecting input noise).

#### 2.1.4 Phase‐randomisation

We repeated HRF estimation on null data by phase‐randomising the BOLD signals (Handwerker et al., 2012) to then obtain the correlation between estimated and phase‐randomised BOLD data at every iteration. We used these correlations to construct a null distribution to test whether correlations obtained from the original data were statistically different than those from the null distribution. A null distribution was constructed for each BOLD signal as well as for each noise regime, that is, combination of confound scaling factors. The statistical tests were Bonferroni corrected for 66 independent tests. Figure S8 depicts how such a null distribution was constructed. Both bootstrapping and the construction of null distributions were performed on Compute Canada clusters. Parallelisation was achieved using GNU Parallel (Tange, 2018).

The entire process of building an HRF tensor, deriving an HRF and spatial–spectral distributions and statistical inference was performed for each of the 66 BOLD signals. In other words, modelling the BOLD signal using LFP data was done separately for each node of the parcellation, resulting in an independent analysis for each node.

## 3 EMPIRICAL DATA

### 3.1 Open‐source datasets

We further apply our methodology to three open‐source EEG‐fMRI datasets, covering resting‐state and task‐based experimental conditions.

Data released by Lioi and colleagues described in (Lioi et al., 2020) contain two sets of EEG‐fMRI data acquired during a motor‐imagery neurofeedback task. Subjects were asked to perform motor‐imagery of clenching their right hand according to a block design, alternating between motor‐imagery and resting blocks. Features derived from the EEG and BOLD‐fMRI acquisitions served as visual feedback cues to drive changes in subject behaviour. This task provides the benefit of limiting sources of artefacts related to motion and muscle tone, in contrast to motor tasks requiring subjects to perform muscle contraction. The first motor‐imagery dataset contains 10 subjects where BOLD images were acquired with a repetition time of 2 s over a total acquisition time of 6 min and 40 s, whereas the second dataset contains 16 subjects where the repetition time was set to 1 s over a total acquisition time of 5 min and 20 s.

Data made publicly available by Deligianni and colleagues contains simultaneous EEG‐fMRI acquisitions of 16 subjects at resting‐state (Deligianni et al., 2014, 2016). The repetition time for BOLD data is 2.16 s over a total acquisition time of 11 min and 20 s. Upon loading EEG data within EEGLAB, it was made apparent that two different EEG electrode layouts were used during data collection: both layouts are displayed in Figure S9. We decided to retain the 11 subjects associated with the first layout (Figure S9a) to simplify our analysis.

In the current article, we focus on Lioi and colleagues' second motor imagery dataset (16 subjects, TR = 1 s) and the resting‐state dataset from Deligianni and collaborators (11 subjects, TR = 2.16 s). Results for these two datasets are thus included within the main body of this article. Results for the first motor‐imagery dataset (10 subjects, TR = 2 s) are shown in the Supporting Information for completeness.

### 3.2 Pre‐processing

Recording EEG within the MRI environment using Echo Planar Imaging sequences gives rise to significant artefacts, the most commonly described throughout the literature being the gradient artefact and the ballistocardiogram (BCG) artefact (Abreu et al., 2018). All three datasets contain versions of EEG data where both gradient and BCG artefacts have been removed using common denoising pipelines, along with standard EEG pre‐processing steps such as down‐sampling. Furthermore, we re‐referenced EEG data to the common average reference allowing us to re‐insert the FCz channel which was used as reference during recording. We also notch‐filtered the data to eliminate remaining artefactual signals. For both motor‐imagery datasets, residual power in the 30–35 Hz and 44–46 Hz ranges was removed. Residual line noise around 50 Hz was removed from resting‐state data. These latter pre‐processing steps were performed in EEGLAB (Delorme & Makeig, 2004) for all three datasets.

The raw BOLD data were pre‐processed using FSL (Jenkinson et al., 2012) for all three datasets. Our pipeline included common pre‐processing steps such as brain‐extraction using BET, deleting initial BOLD volumes, high pass filtering at a 100 ms cut‐off, McFLIRT motion correction, slice‐timing correction and 5 mm FWHM spatial smoothing using FEAT. Functional data were registered to structural data using FLIRT's boundary‐based registration (BBR) which uses white‐matter (WM) segmentations to align functional and structural volumes (Greve & Fischl, 2009). Structural data were registered to the MNI152 2 mm standard space using NFLIRT with 12 degrees‐of‐freedom and a 10 mm warp resolution.

Many pre‐processing approaches have been suggested for further ridding BOLD‐fMRI data from head motion effects and systemic physiological artefacts. For each subject, we employed a strategy very similar to anatomical CompCor (Muschelli et al., 2014) which consists of using WM eigenvectors for removing motion‐ and physiology‐related sources of structured noise within a regression model (Kassinopoulos & Mitsis, 2022). Specifically, the WM segmentation obtained from the BBR registration routine was used as an overlay map to extract 10 eigenvectors explaining the most variance using FSL's *fslmeants* routine. These 10 WM eigenvectors were used as regressors within a GLM model to fit these regressors to all brain voxel time‐series using *fsl_glm*. The residuals of this GLM‐based WM regression, considered as the clean BOLD data, were used for further analysis.

#### 3.2.1 Dimensionality reduction

We performed dimensionality reduction via multi‐subject blind source separation (BSS) for both EEG and BOLD‐fMRI data. This reduction reduces the number of possible combinations between EEG and BOLD time‐courses, while also providing a succinct representation of the data at hand. From a methodological viewpoint, group‐level ICA analyses come with certain challenges when it comes to comparing results across subjects or deriving group statistics. Temporal concatenation followed by dual‐regression is a frequently adopted approach for multi‐subject ICA analysis of BOLD‐fMRI data (Nickerson et al., 2017). This is the approach we have adopted in this study. Accordingly, we executed ICA on BOLD data of spatially normalised subjects which were concatenated in the temporal dimension. We set the number of output components to 25 as this was empirically observed to provide the desired spatial coverage and separation of networks.

This multi‐subject ICA decomposition of BOLD‐fMRI data returned spatial maps and their associated time‐courses (Figure 2b). The group ICA spatial maps were used as spatial regressors for all subjects, returning one subject‐specific time‐course for each group spatial map. We limited dual regression to its first step (i.e. spatial regression) as only subject‐specific time‐series were necessary for the remainder of the analysis. Group ICA and the spatial regression step of dual regression were executed with FSL MELODIC and FSL's *dual_regression* routine, respectively.

Although the temporal concatenation approach followed by dual‐regression is commonplace for group BOLD‐fMRI studies, the appropriateness of similar approaches has required more clarification for multi‐subject EEG studies (Huster et al., 2015). We adopted recommendations from Lio and Boulinguez (2013) of substituting ICA decompositions based on higher‐order statistics for second‐order statistics‐based decompositions. Specifically, we performed the second‐order blind identification (SOBI) algorithm (Belouchrani et al., 1997) on concatenated EEG data for performing group‐level BSS. Compared to ICA based on higher‐order statistics, SOBI is argued in Lio and Boulinguez (2013) to be more specific to the spectral content of the data when performing its decomposition. Moreover, it is also thought to be more robust to inter‐subject variability of the mixing process that underlies the electrical field propagation from neuronal dipoles to EEG sensors. Performing SOBI on concatenated EEG data returns group‐level spatial topographies, similarly to group ICA for BOLD‐fMRI. Group‐wide scalp topographies are then used as spatial regressors to derive subject‐specific time‐courses for each SOBI component (Figure 2a), similarly to dual regression for BOLD‐fMRI. Multi‐subject SOBI was executed in EEGLAB, generating a total of 63 EEG components, which represents the maximum allowable number of components.

Out of the total of 63 EEG and 25 BOLD neuronal components, a subset of components was manually selected for further investigation. For all datasets, initial selection of EEG components was based on identifying scalp topographies associated with neuronal activity in the literature. Time‐series for SOBI components were visually inspected to validate that components reflected neuronal activity. The manual selection of BOLD components followed the same reasoning that of EEG data, although it was more conservative to make our analysis more manageable. We have restricted our search to four components for each of the motor‐imagery datasets of Lioi and colleagues, and three components for the resting‐state data of Deligianni and colleagues. Despite limiting the number of selected components, we were capable of retrieving relevant networks covering motor, somatosensory and premotor areas for motor‐imagery data as well as well‐known resting‐state networks (RSN) for resting‐state data. The Juëlich Histological Atlas was used for identifying functional regions within the spatial maps of BOLD components and thus guiding our manual selection.

#### 3.2.2 HRF tensor and CPD decomposition

The outputs of interest from group‐level BSS were the subject‐specific EEG and BOLD time‐courses. For each subject, multi‐tapering (Babadi & Brown, 2014) was applied to the time‐course of each EEG SOBI component to obtain their time‐frequency representations (Figure 2c‐i). In this way, amplitude dynamics are obtained for each band centred at selected frequencies across each SOBI component. The selected centre frequencies were [4, 6, 8, 10, 12, 16, 20, 24, 28, 32, 40, 50] Hz, covering the theta to low‐gamma EEG frequency range. We used MNE Python (Gramfort et al., 2013) for multi‐tapering with default settings, that is, 7 cycles per wavelet and a time‐bandwidth product of 4. It is noteworthy that the centre frequencies listed above include frequencies which have been filtered during pre‐processing as mentioned in Section 3.2)—Pre‐processing of the Methods section. Specifically, the 32 Hz and 50 Hz centre frequencies were suppressed when bandstop filtering within the 30–35 Hz range for motor‐imagery data as well as notch filtering of line noise at 50 Hz for resting‐state data. The inclusion of these filtered frequencies was mainly allowed for the sake of completeness. For instance, although we have retained CPD components which contain non‐zero weightings at 32 Hz and 50 Hz, the larger downstream objective is to adequately model the BOLD signal. Spurious weightings at 32 Hz or 50 Hz were not found to significantly affect modelling performance in cases where other frequencies with stronger weightings dominated.

After down‐sampling amplitude signals to the sampling rate of BOLD data, a SSRF was estimated between each amplitude signal—for each EEG SOBI component and frequency—and the time‐course of a given BOLD group‐ICA component (Figure 2c‐i). These SSRFs are stored into a third‐order HRF tensor for each subject, spanning dimensions of time, SOBI components and frequencies (Figure 2c‐ii). For lack of a better term, we refer to the dimension covering different SOBI components as the dimension of *space* since the EEG spatial topographies of SOBI components are used to represent these components. We also performed rescaling of each third‐order HRF tensor as explained in Supplementary Methods 1—Rescaling the HRF tensor using soft‐thresholding.

The size of the temporal, spatial and spectral dimensions was implicitly constrained by the length of the estimated SSRFs, number of selected EEG SOBI components and number of selected frequencies, respectively. The third‐order HRF tensors from each subject were then combined, forming a fourth‐order HRF tensor spanning dimensions of time, SOBI components, frequencies and subjects (Figure 2c‐ii). To pre‐process this tensor prior to CPD (Bro & Smilde, 2003), we performed scaling across the subject dimension and centering across the frequency dimension. The fourth‐order HRF tensor was then decomposed into a rank‐1 representation using CPD, giving rise to a dominant HRF as well as spatial, spectral and subject‐level distributions (Figure 2c‐iii). For all three empirical datasets, we executed our analysis once with the number of CPD components set to 1 and an additional time with the number of CPD components set to 2. Outputting multiple CPD components allows us to extract more EEG spatial and spectral correlates of a given BOLD signal. Outputting multiple CPD components is similar to outputting multiple PCA components, each component serving as a basis to describe the data at hand.

Similar to our analysis based on simulated data, SSRFs were estimating using functional basis expansion based on spherical Laguerre functions. We fixed the number of basis functions to 3 which provides balance between flexibility and robustness as observed empirically. However, the decay‐rate parameter also needs to be specified, as well as the correlation percentile for soft‐thresholding the correlation matrix used for rescaling and sparsifying the HRF tensor (readers can refer to Figure S5 for rescaling of HRF tensor). Thus, for each IC, we performed a parameter sweep in order to find optimal decay‐rate parameters and correlation percentiles used for thresholding. The optimal values for the parameters were selected as those that returned the highest single‐subject correlation between the ensuing BOLD signal and its estimate.

Values for decay‐rate parameters during the parameter sweep varied between 0.8 and 4.8 in leaps of 0.2 whereas percentiles of 0, 25, 50 and 75 were evaluated for selecting the optimal correlation percentile. Parameter sweeps were performed independently for the two cases examined, that is, when the number of CPD components was set to 1 and 2, respectively.

#### 3.2.3 Jackknifing and statistical inference

To evaluate the stability of CPD applied to the fourth‐order HRF tensors, we adopted a jackknifing approach (Young et al., 1996). This method consists of deleting datapoints from EEG and BOLD data prior to constructing the HRF tensor. CPD was then applied to the truncated data to return the estimated HRF as well as the corresponding spatial, spectral and subject‐level distributions. The jackknife procedure involved deletion of 2 datapoint (delete‐2 jackknife) and was repeated 1000 times to obtain median values and 5th/95th percentiles of these HRFs and their related distributions.

An important aspect of CPD to consider for obtaining jackknife estimates is the sign ambiguity of the decomposition. For example, the frequency distributions of jackknife iterations *i* and *j* may be mirror copies of each other in terms of the sign of the elements of the distributions (i.e. of the weightings). Hence, in such a case, one would need to account for this sign ambiguity before computing median and percentile values of the frequency distribution. Another issue is the permutation ambiguity when CPD returns multiple components rather than just one. For example, it may be the case that the first component of jackknife iteration *i* corresponds to the second component of iteration *j*. For computing proper jackknife statistics, it becomes necessary to adequately group components across iterations. To account for the permutation ambiguity when 2 components are requested from CPD and to correct for the sign ambiguity, we used the *cpderr*.*m* function from the *Tensorlab* MATLAB package (Vervliet et al., 2016) by running the MATLAB Engine API for Python. It should be noted that only the sign of the scaling factors output from *cpderr*.*m* were used to correct for sign ambiguity, discarding the magnitude of these scaling factors.

Similar to the compound LFP signals derived from simulated data, we derived a so‐called compound EEG signal by weighing the EEG amplitude signals with the spatial and spectral via the N‐mode product along spatial and spectral modes. The compound EEG signal was then convolved with the estimated HRF to obtain an estimate of the BOLD signal (Figure 2c‐iii,iv). The estimated BOLD signal was correlated with the BOLD ICA component under consideration, as obtained from the experimental measurements. The resulting correlation coefficient served to quantify the goodness‐of‐fit of the BOLD signal estimate derived from EEG data, CPD outputs and N‐mode products. These correlation coefficients were compared against a null distribution as described earlier for our analysis using simulated data in Section 2.1.4)—phase‐randomisation from Methods. Readers may also refer to Figure S8 for a visual depiction of how null distributions were constructed, enabling the statistical inference procedure.

## 4 RESULTS

### 4.1 Simulated data

**FIGURE 3:**
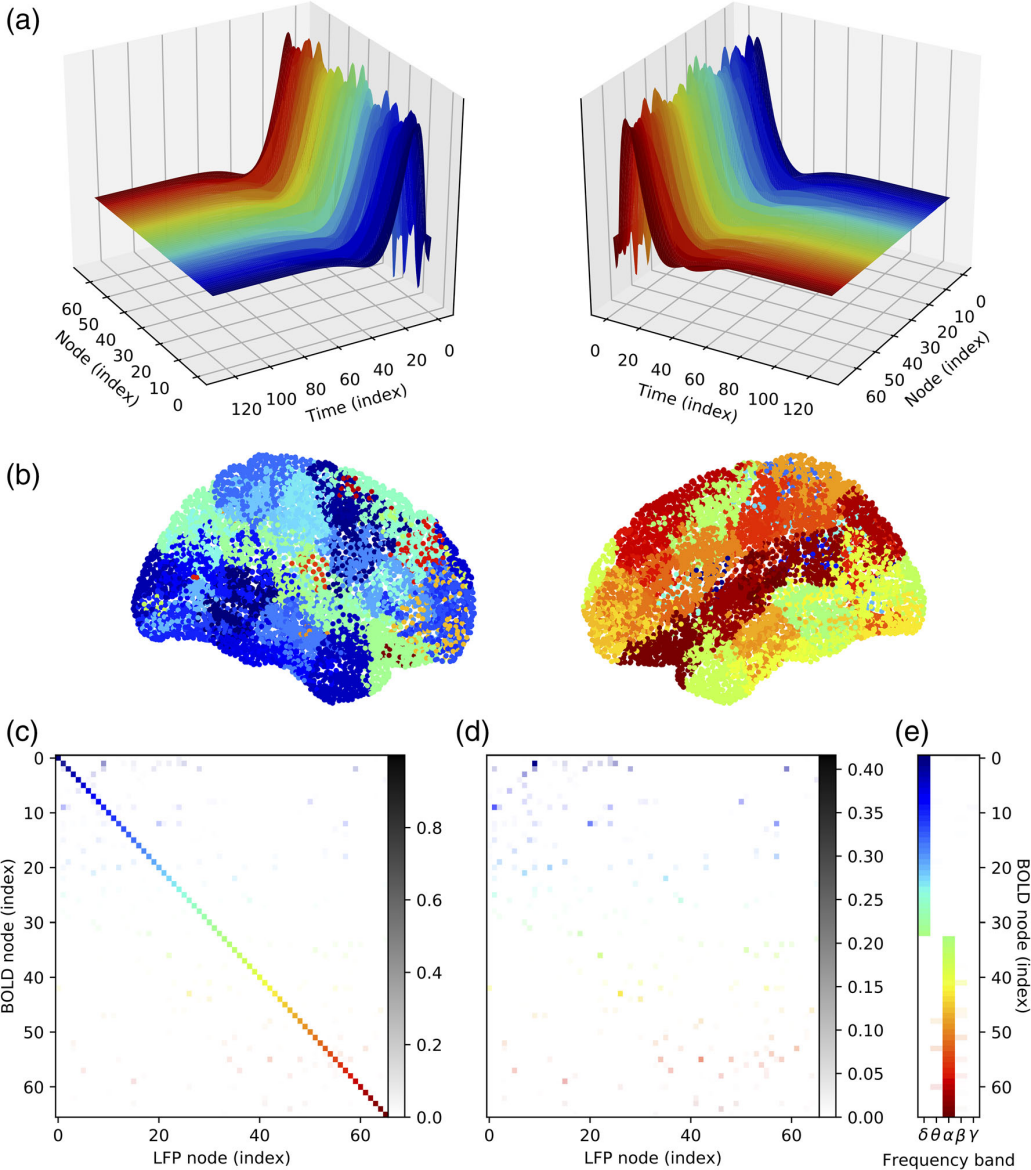
Overall results for all nodes using simulated data. (a) HRFs are viewed from different angles. (b) Cortical nodes of Desikan–Killiany parcellation, colour‐coded. (c) Spatial distributions, where colours map to cortical nodes (b) and transparency reflects the weightings. Grayscale bar illustrates correspondence between transparency and weighting. This matrix is best read strictly row‐wise, where CPD of the HRF tensor associated with a given ‘BOLD node’ results in a spatial distribution involving multiple ‘LFP nodes’ to varying degrees. (d) Same as (c), where the diagonal was removed and transparencies rescaled. (e) Spectral distributions

Figure 3 provides an overview of the estimated HRFs and spatial–spectral distributions resulting from our analysis applied to simulated data. Results are colour‐coded by the nodes of the Desikan–Killiany parcellation (Figure 3b). Estimates of node‐wise HRFs (Figure 3a), spatial distributions (Figure 3c,d) and spectral distributions (Figure 3e) are shown for all nodes. For results shown in Figure 3c–e, the weightings for different LFP nodes (Figure 3c,d) and frequency bands (Figure 3e) are represented by the transparency of the entry rather than by colour. Figure 3c,d differ only in the weightings for diagonal elements which are preserved in Figure 3c and removed in Figure 3d. Both subfigures contain grayscales to display the correspondence between transparency and weightings. Transparency was rescaled in Figure 3d for visibility, as can be observed by comparing the grayscales of Figure 3c and Figure 3d. It is noteworthy that Figure 3e adequately captures the frequency bands associated with each node. Namely, the first half of nodes shows weightings in the δ bands as expected for oscillators tuned at 2 Hz, whereas the second half shows weightings in the α band as expected for oscillators tuned at 10 Hz although with some occasional leakage into adjacent frequency bands.

For an example of more detailed results for a single node, readers are invited to consult Figure S10 and related methodology in Supplementary Methods 3–4 included in the Supporting Information. Briefly, we show that our CPD‐based method was able to derive proper spatial and spectral distributions. Moreover, the estimated HRF derived from CPD is in good agreement with reference HRFs, where the latter are used as proxies for the ground‐truth. Two different parametrisations of the balloon model were used to generate HRFs: one which generates a post‐stimulus undershoot as shown in Figure S10d and one which does not generate such an undershoot, as shown in Figure S10d2. We also demonstrate, in Figure S11, the improved modelling capacity of simulated BOLD signals when using an HRF derived from our CPD‐based method compared to the modelling capacity when using the canonical HRF.

**FIGURE 4:**
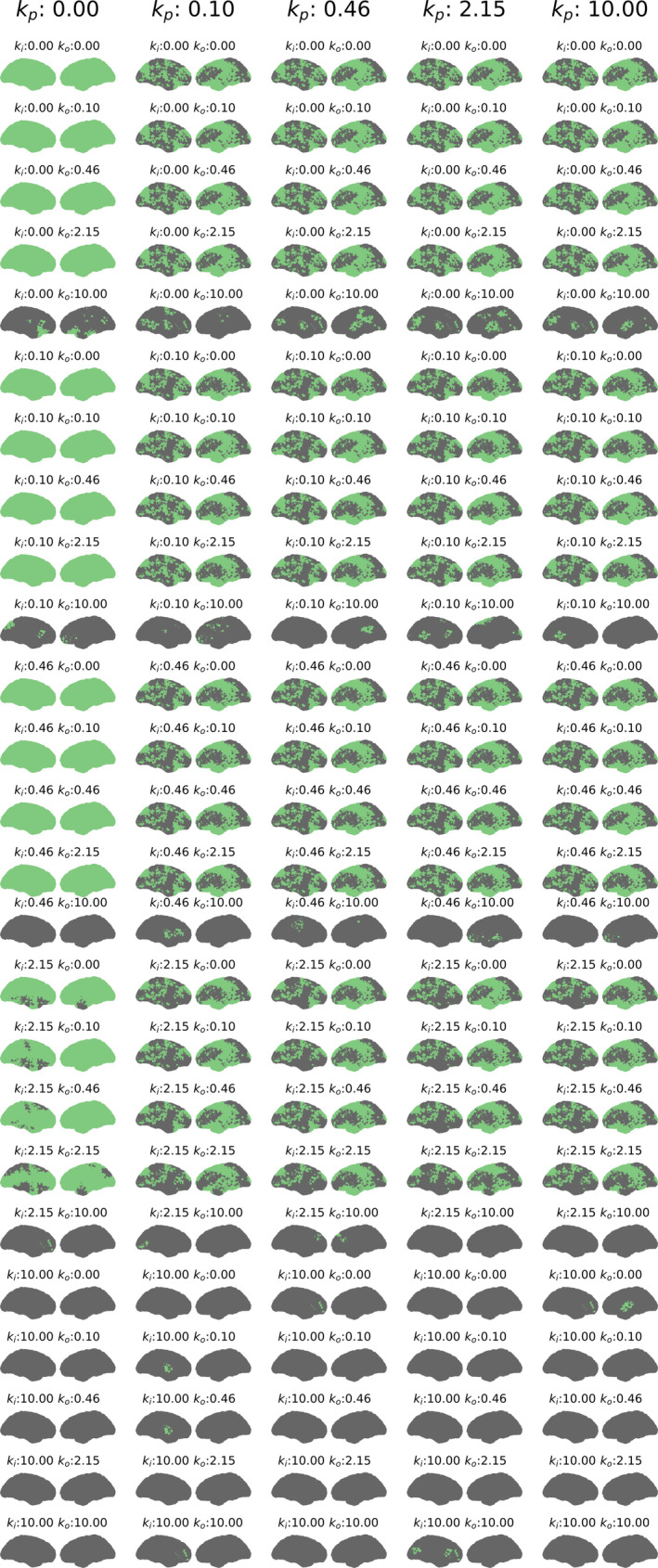
Statistical inference, based on correlations between estimated and real BOLD signals. Regions rejecting the null hypothesis are highlighted in green. Regions failing to reject the null hypothesis are highlighted in dark grey. Results are shown for different values of the scaling coefficients $k_{p}$ (SLFOs ‐ fixed across each column), $k_{i}$ (input measurement noise) and $k_{o}$(output measurement noise). The coefficients $k_{p},k_{i}$ and $k_{o}$ were individually set to one of the five following values: 0, 0.1, 0.46, 2.15 and 10. This array of possible values thus results in $5^{3}=125$ combinations. This figure suggests that our analysis is robust to output measurement noise and fairly robust to input measurement noise, although highly susceptible to physiological confounds. Indeed, the scaling coefficients of input ($k_{i}$) and output ($k_{o}$) measurement noise requires minimal values of 10 and 2.15, respectively, to show any effect on results. On the other hand, the slightest gain in SLFOs (i.e. $k_{p}\geq 0.10$) induces spatial distributions of regions where the null hypothesis is not rejected

Figure 4 shows results for statistical inference of the goodness‐of‐fit of BOLD signal estimates using correlations (Figure S8 for methodology). Results are shown for all noise regimes, that is, all combinations of the confound scaling coefficients $k_{p},k_{i}$ and $k_{o}$ which respectively scale physiological, input measurement and output measurement noise (Figure S6). For every combination of scaling coefficients, a spatial topography for both hemispheres is shown of nodes either rejecting (green) or failing to reject (dark gray) the null hypothesis. The null hypothesis posits that correlations obtained from phase‐randomised BOLD data are similar to the correlation obtained from the original BOLD signal. Hence, the null hypothesis was tested for each node. It can be seen that whereas nonzero $k_{i}$ and $k_{o}$ translate into nodes failing to reject the null hypothesis in a uniform fashion across nodes, nonzero $k_{p}$ translates into structured patterns of nodes failing to reject the null hypothesis. These latter structured patterns are expected, considering our method of first deriving node‐specific scaling values from correlation maps before scaling all SLFOs uniformly using $k_{p}$.

### 4.2 Empirical data

Results in this section are focused on the second motor‐imagery dataset from Lioi et al. (2020) and the resting‐state dataset from Deligianni et al. (2014, 2016). Hence, mention of the motor‐imagery dataset in the following section refers by default to the second motor‐imagery dataset. Results for the first motor‐imagery dataset are presented in the Supporting Information.

**FIGURE 5:**
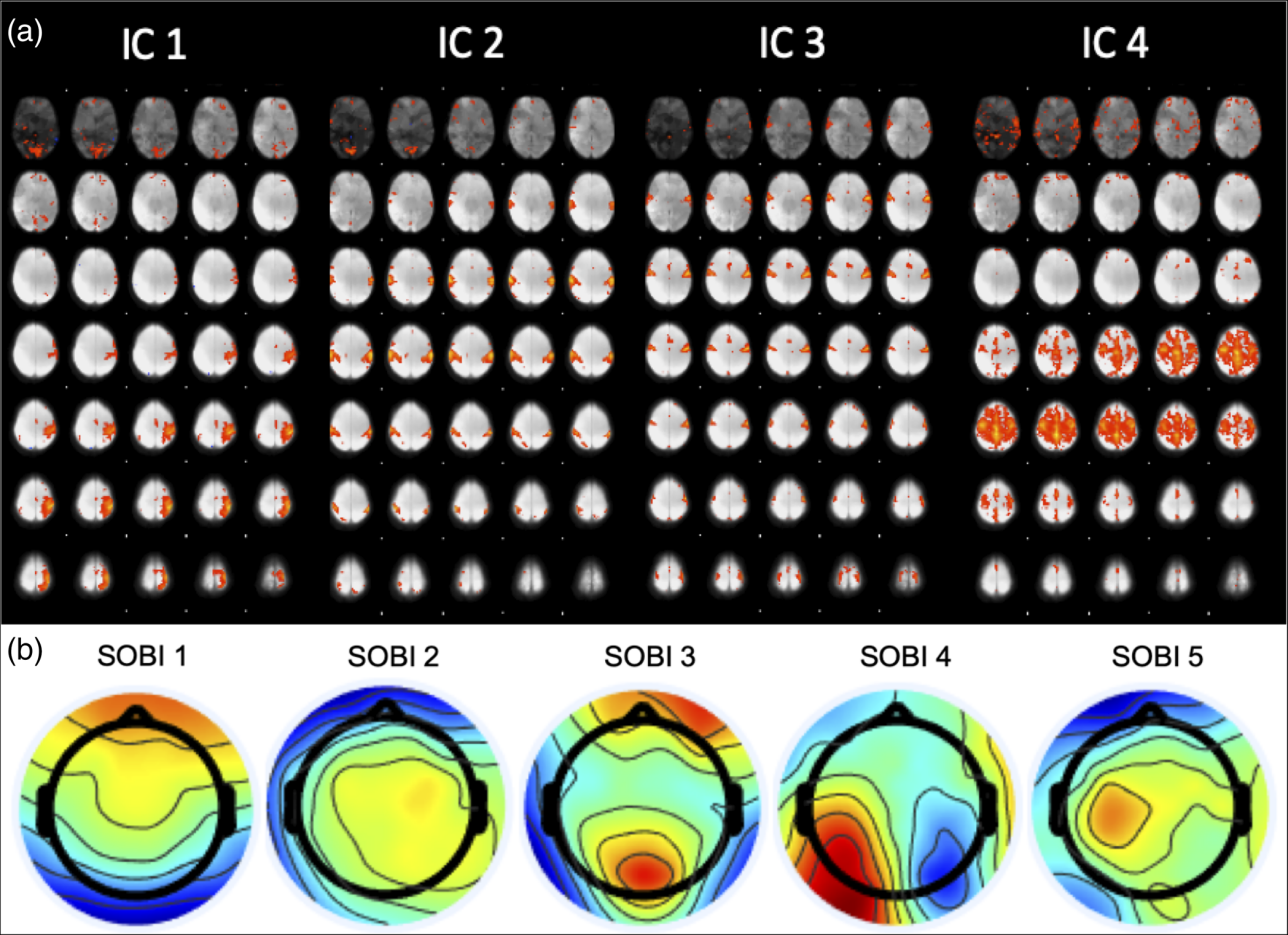
BOLD independent components (IC) in panel a and scalp topography of EEG SOBI components in panel b for motor‐imagery data. Main spatial coverage for BOLD IC1—left primary motor and somatosensory cortices; IC2—bilateral primary somatosensory cortices and inferior parietal lobules; IC3—bilateral primary motor and somatosensory cortices; IC4—bilateral premotor cortex. Regions of BOLD ICs determined using Juëlich histological atlas

**FIGURE 6:**
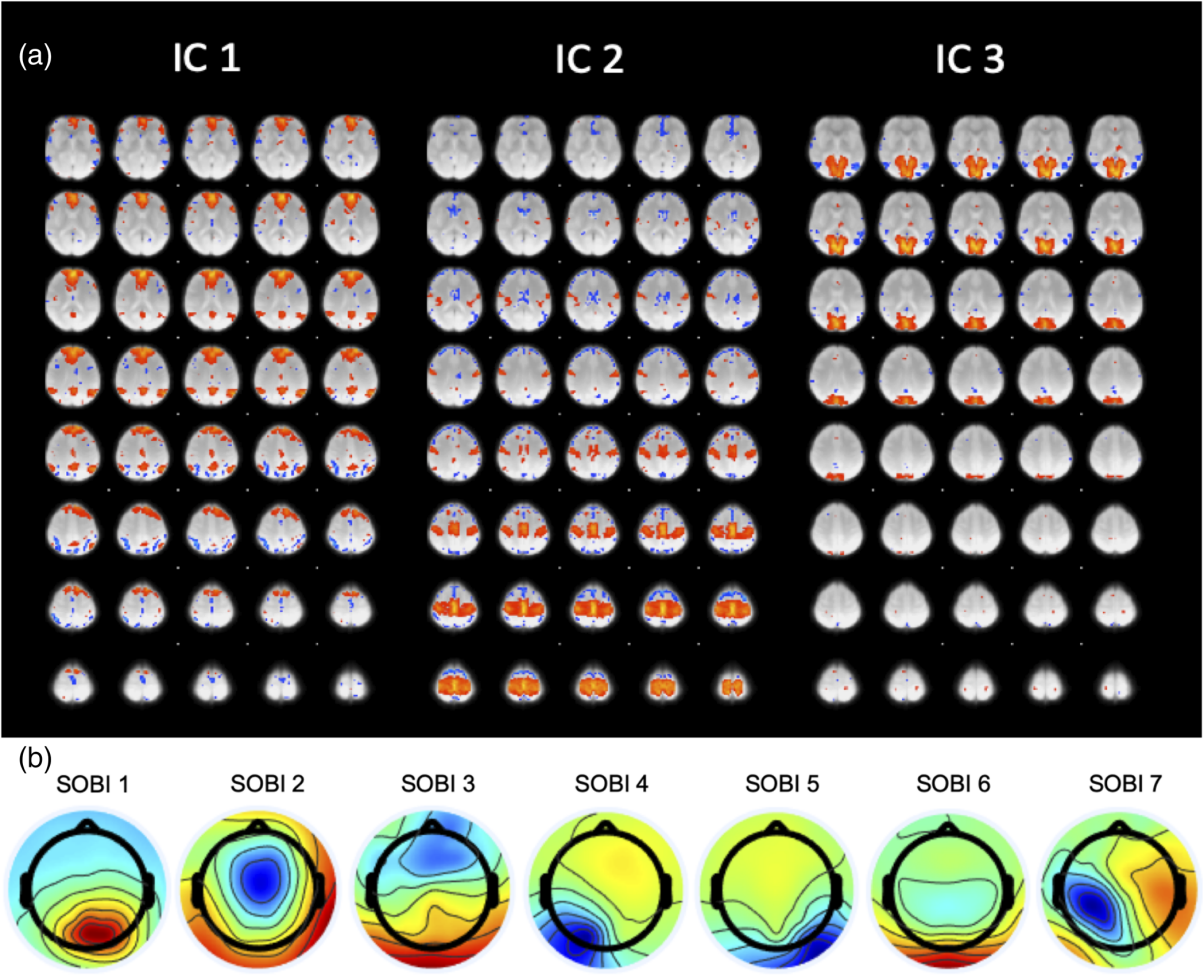
BOLD independent components (IC) and scalp topography of EEG SOBI components for resting‐state data. Corresponding RSN for IC1 is default mode network (DMN); for IC2 is somatosensory network (SN); for IC3 is visual network (VN)

BOLD spatial maps obtained from group ICA are shown in Figures 5a and 6a for the motor‐imagery and resting‐state datasets, respectively. The manual selection of group ICA components for the motor‐imagery data reflects the task at hand and thus explains the dominantly motor and somatosensory coverage (Figure 5a). On the other hand, components for resting‐state data were selected based on well‐known resting‐state networks (RSNs) such as the Default Mode Network (DMN), Somatosensory Network (SN) and Visual Network (VN) corresponding to BOLD ICs 1, 2 and 3, respectively (Figure 6a). Alongside the BOLD group spatial maps, we also show group EEG SOBI scalp topographies in Figure 5b for the motor‐imagery dataset and Figure 6b for the resting‐state dataset. Group SOBI components were chosen based on their corresponding scalp topographies and spectral contents. For both datasets, SOBI topographies show spatial coverage in occipital, sensorimotor and frontal regions (Figures 5b and 6b).

### 4.3 Motor‐imagery dataset

**FIGURE 7:**
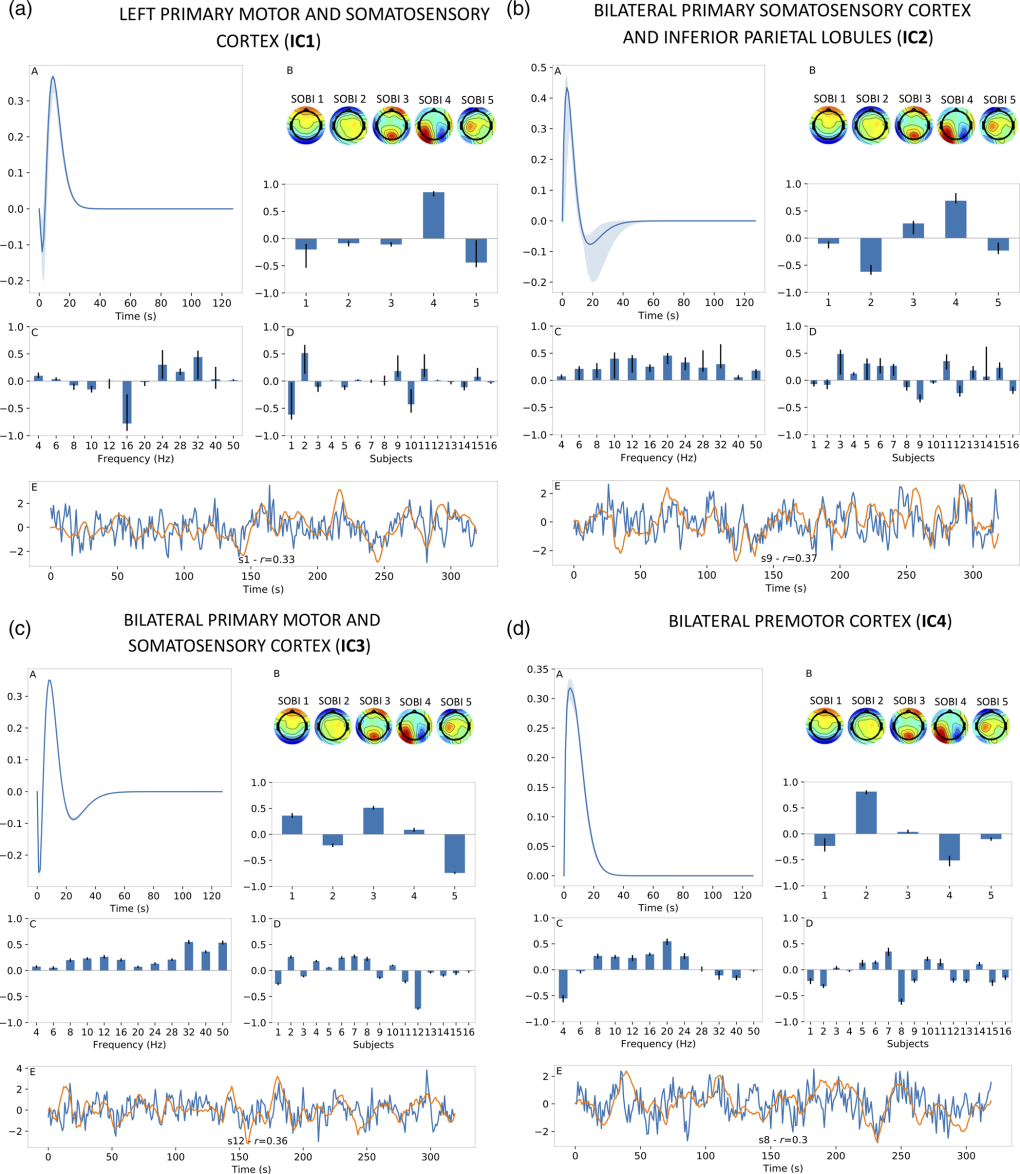
Results for motor‐imagery data when using 1 CPD component. Figure a–d shows results for different BOLD independent components (ICs, Figure 5a). For each IC, estimated HRF shown in panel A, EEG SOBI topographies (Figure 5b) and spatial distribution in panel B, frequency distribution in panel C, subject distribution in panel D and BOLD signal estimate (orange) alongside real BOLD signal (blue) in panel E. For panel E, results were presented for subject with the highest correlation coefficient (*r*) between BOLD signal estimate and real BOLD signal. The vertical axis of each subfigure bears arbitrary units. In text, for example, Figure 7a‐B refers to Panel B of Figure 7a.

Our proposed analysis was performed on each IC, returning in each case an HRF estimate as well as the corresponding spatial, spectral and subject distributions. Results, when the number of CPD components was set to 1, are displayed for the motor‐imagery data in Figure 7a–d for ICs 1 through 4, respectively. Each figure shows the estimated HRF (panel a), group EEG SOBI topographies along with their spatial distribution (panel b), spectral distribution (panel c), subject distribution (panel d) and real time‐series of the BOLD IC (blue trace) and its estimate (orange trace) for the subject displaying the highest correlation (panel e). Alongside these results are confidence intervals bounded at the 5th and 95th percentiles based on the delete‐2 jackknife procedure. Overall, the extracted HRFs exhibited dynamics in agreement with previous studies (Devonshire et al., 2012; Taylor et al., 2018), whereby the function peaks around 5–10 s before either stabilising at baseline (Figure 7a‐A and d‐A) or transitioning into an undershoot before returning to baseline (Figure 7b‐A, c‐A). HRFs in Figure 7a‐A,c‐A also demonstrate a strong initial dip, although this may be due to the shape of the spherical Laguerre basis functions. The shape of the frequency distributions was found to be broadband and smooth across frequencies (Figure 7b‐C–d‐D) in most cases, although this was not always the case (Figure 7a‐C). Finally, we can observe correlations between BOLD signal and its estimate reaching maximal single‐subject values between 0.30 and 0.37 for different ICs (Figure 7a‐E–d‐E). These correlation values are satisfactory from a qualitative standpoint and mirror the ability of BOLD signal estimates to model slower variations in BOLD dynamics as observed from visual inspection. A more quantitative assessment is presented later on and illustrated in Figure 11.

**FIGURE 8:**
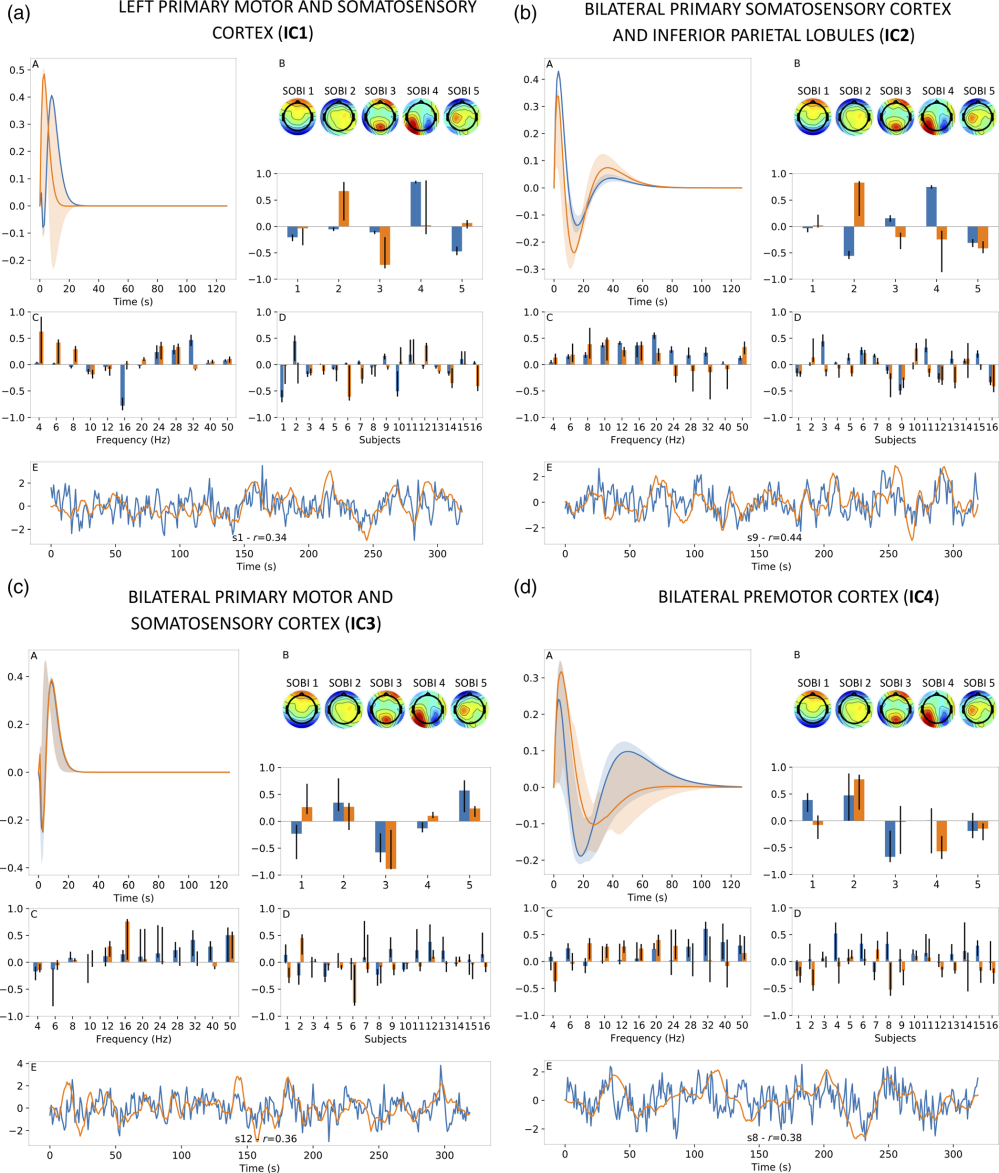
Results for motor‐imagery data when using 2 CPD components. Figure a–d shows results for different BOLD independent components (ICs, Figure 5a). For each IC, estimated HRF is shown in panel A, EEG SOBI topographies (Figure 5b) and spatial distribution in panel B, frequency distribution in panel C, subject distribution in panel D and BOLD signal estimate (orange) alongside real BOLD signal (blue) in panel E. For panel E, results were presented for subject with the highest correlation coefficient (*r*) between BOLD signal estimate and real BOLD signal. The vertical axis of each subfigure bears arbitrary units. In text, for example, Figure 8a‐B refers to Panel B of Figure 8a.

Results when setting the number of CPD components to 2 are displayed for motor‐imagery data in Figure 8a through Figure 8d for ICs 1 through 4, respectively. Observations made earlier for results displayed in Figure 7 generally hold for those shown in Figure 8 as well. One obvious difference is the addition of a second component which is colour‐coded in orange in Panels A–D of Figure 8a–d. In the case where two components were returned by CPD, we obtained two compound EEG signals which, once convolved with their respective HRFs, each provide an estimate of the BOLD signal for a given IC. To obtain a single estimate of the BOLD signal as shown in Panels E, we fit both estimates to the BOLD signal via linear regression. This linear regression step was also applied during jackknifing iterations and the construction of null distributions.

According to Figure 8, having two regressors to model the same BOLD signal translated into better BOLD signal estimates for some ICs. For instance, the maximal correlation for IC2 grew from 0.37 when using 1 CPD component (Figure 7b‐E) to 0.44 when using 2 CPD components (Figure 8b‐E). Alongside this improved performance for IC2, its CPD outputs show expected general characteristics (HRF shape, smooth and broadband frequency distributions, etc.) and relatively narrow confidence intervals. Otherwise, improvements were not as marked for other ICs although still noticeable, as is the case for IC4 where the maximal correlation increased from 0.30 to 0.38 (Figure 7d‐E vs. Figure 8d‐E) although at the expense of wider confidence intervals for the CPD outputs. On the other hand, IC2 and IC3 did not exhibit a clear benefit from adding a second CPD component (i.e. Figure 7b vs. Figure 8b and Figure 7c vs. Figure 8c, respectively).

Table 1 lists the decay‐rate parameters as well as percentile values used for soft‐thresholding the HRF tensor. These values were chosen based on the parameter sweep described in the Methods section.

**TABLE 1:** Decay‐rate parameter and percentile selected by the parameter sweep for each independent component (IC) for motor‐imagery data

Independent component (IC)	Decay‐rate parameter	Percentile
1 CPD component		
IC1	1.2	75
IC2	2.2	25
IC3	2.2	0
IC4	1.4	0
2 CPD components		
IC1	1.0	50
IC2	3.2	25
IC3	1.0	25
IC4	4.8	0

*Note*: Shown for the number of canonical polyadic decomposition (CPD) components set to 1 and 2.

The Supporting Information contains results pertaining to the second motor‐imagery dataset not included in the main body of this article. In brief, BOLD group ICA components shown in Figure S12a cover much of the same motor and somatosensory areas. Using 1 CPD component yields HRFs with sensible shapes, broadband frequency distributions and high maximal correlations between BOLD signal and BOLD signal estimate in the range of 0.35–0.42 (Figure S13). A comparison of results shown in Figures S13 and S14 suggests that adding a second CPD component does not significantly provide additional information, resulting in a fewer number of cases where the null hypothesis is rejected as exhibited in Figure S15.

### 4.4 Resting‐state dataset

**FIGURE 9:**
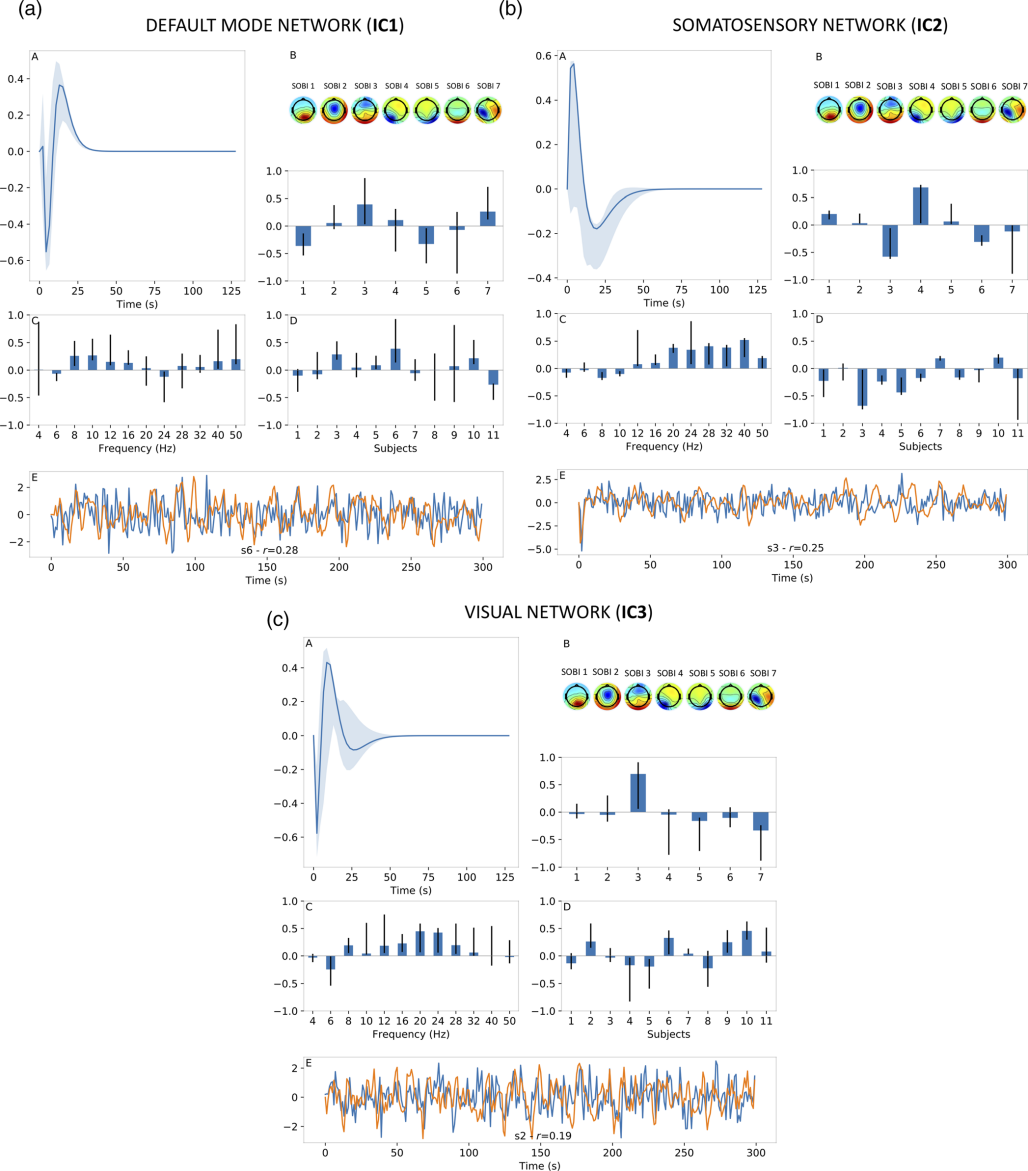
Results for resting‐state data when using 1 CPD component. Figure a–c shows results for different BOLD independent components (ICs, Figure 6a). For each IC, estimated HRF shown in panel A, EEG SOBI topographies (Figure 6b) and spatial distribution in panel B, frequency distribution in panel C, subject distribution in panel D and BOLD signal estimate (orange) alongside real BOLD signal (blue) in panel E. For panel E, results were presented for subject with the highest correlation coefficient (*r*) between BOLD signal estimate and real BOLD signal. The vertical axis of each subfigure bears arbitrary units. In text, for example, Figure 9a‐B refers to Panel B of Figure 9a.

**FIGURE 10:**
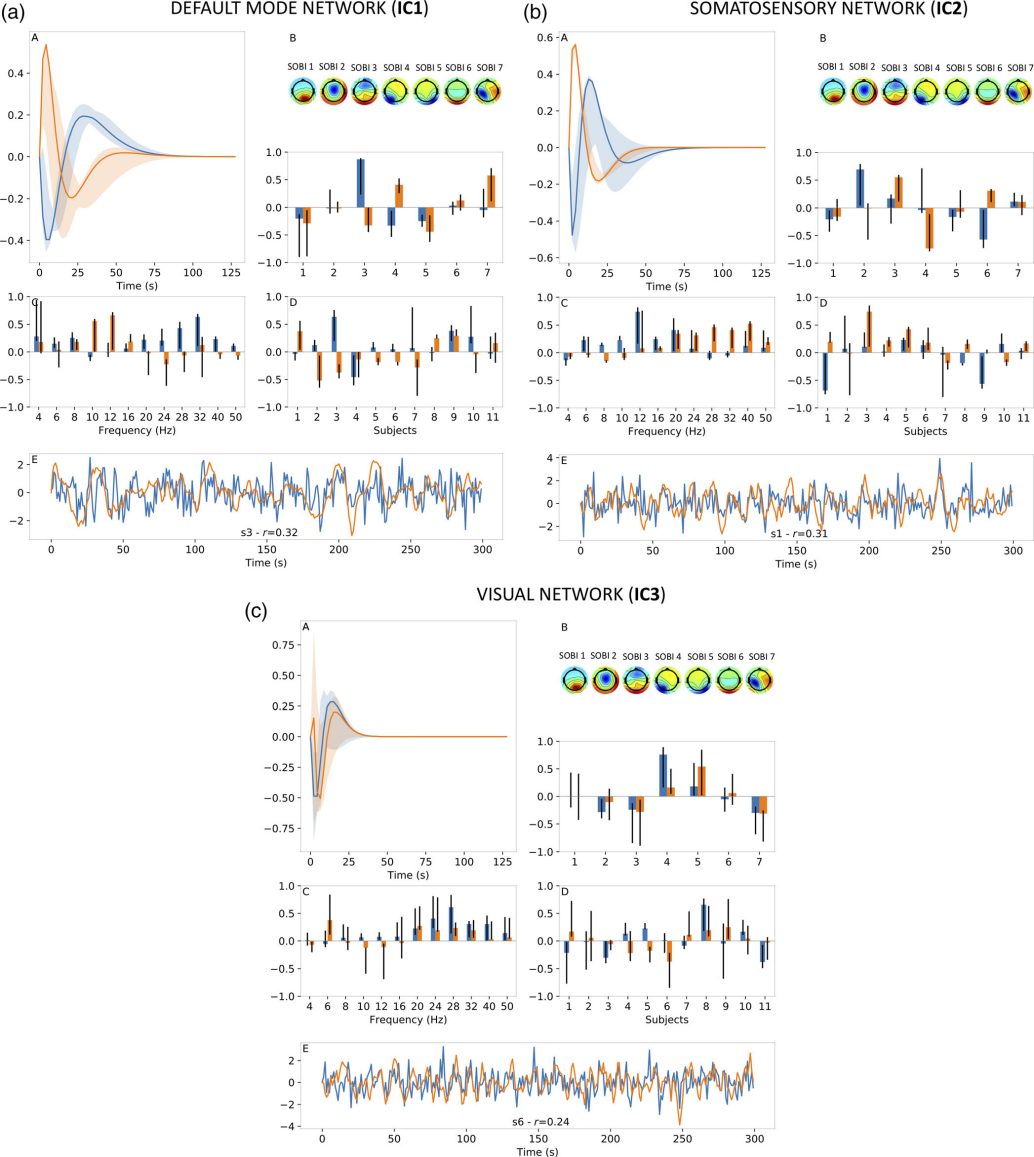
Results for resting‐state data when using 2 CPD component. Figure a–c shows results for different BOLD independent components (ICs, Figure 6a). For each IC, estimated HRF shown in panel A, EEG SOBI topographies (Figure 6b) and spatial distribution in panel B, frequency distribution in panel C, subject distribution in panel D and BOLD signal estimate (orange) alongside real BOLD signal (blue) in panel E. For panel E, results were presented for subject with the highest correlation coefficient (*r*) between BOLD signal estimate and real BOLD signal. The vertical axis of each subfigure bears arbitrary units. In text, for example, Figure 10a‐B refers to Panel B of Figure 10a.

Homologous results for resting‐state data are shown in Figure 9a–c (1 CPD component) and Figure 10a–c (2 CPD components), alongside Table 2 which lists the selected decay‐rate parameters and percentiles. As before, the HRFs derived from resting‐state data exhibited shapes largely consistent with typical HRF shapes and the frequency distributions were found to be broadband and smooth across frequencies. However, one main difference between results from motor‐imagery data when using 1 CPD component (Figure 7) and those from resting‐state data (Figure 9) are the wider confidence intervals for the latter. This suggests that CPD outputs for resting‐state data exhibited more variability, as assessed by the jackknifing procedure. Another important difference is the quality of BOLD signal estimates, as quantified by the corresponding correlation coefficients. Maximal correlation coefficients from Figure 9a‐E–c‐E for resting‐state ranged between 0.19 and 0.28 compared to 0.30 to 0.38 for the motor‐imagery data (Figure 7a‐E–d‐E).

**TABLE 2:** Decay‐rate parameter and percentile selected by the parameter sweep for each independent component (IC) of resting‐state data

Independent component (IC)	Decay‐rate parameter	Percentile
1 CPD component		
IC1	1.4	0
IC2	2.6	0
IC3	2.2	0
2 CPD components		
IC1	4.0	0
IC2	3.2	0
IC3	1.6	0

*Note*: Values are shown when number of canonical polyadic decomposition (CPD) components set to 1 and 2.

Adding a second CPD component for resting‐state data resulted in improved BOLD signal estimates, which in turn yielded maximal correlation coefficients between 0.24 and 0.32 (Figure 10a‐E–c‐E). Also, whereas adding a second CPD component for motor‐imagery data tended to increase the width of confidence intervals, this was not found to be the case to the same extent for the resting‐state data. In fact, the HRF of the second component shown in Figure 10b‐A in orange is an example of the opposite, where the confidence intervals around this HRF are very tight.

### 4.5 Statistical inference

To assess the statistical significance of the fit between the CPD‐based estimated and real BOLD signals, we constructed null distributions of correlation coefficients by phase randomisation of BOLD data and repeated the procedure of HRF tensor estimation followed by CPD, BOLD signal estimation and correlation coefficient computation. The correlation coefficients obtained from the original data were compared against the null distributions. The BOLD signal estimate was deemed statistically significant if the original correlation coefficient exceeded the 95th percentile of null correlation coefficients.

**FIGURE 11:**
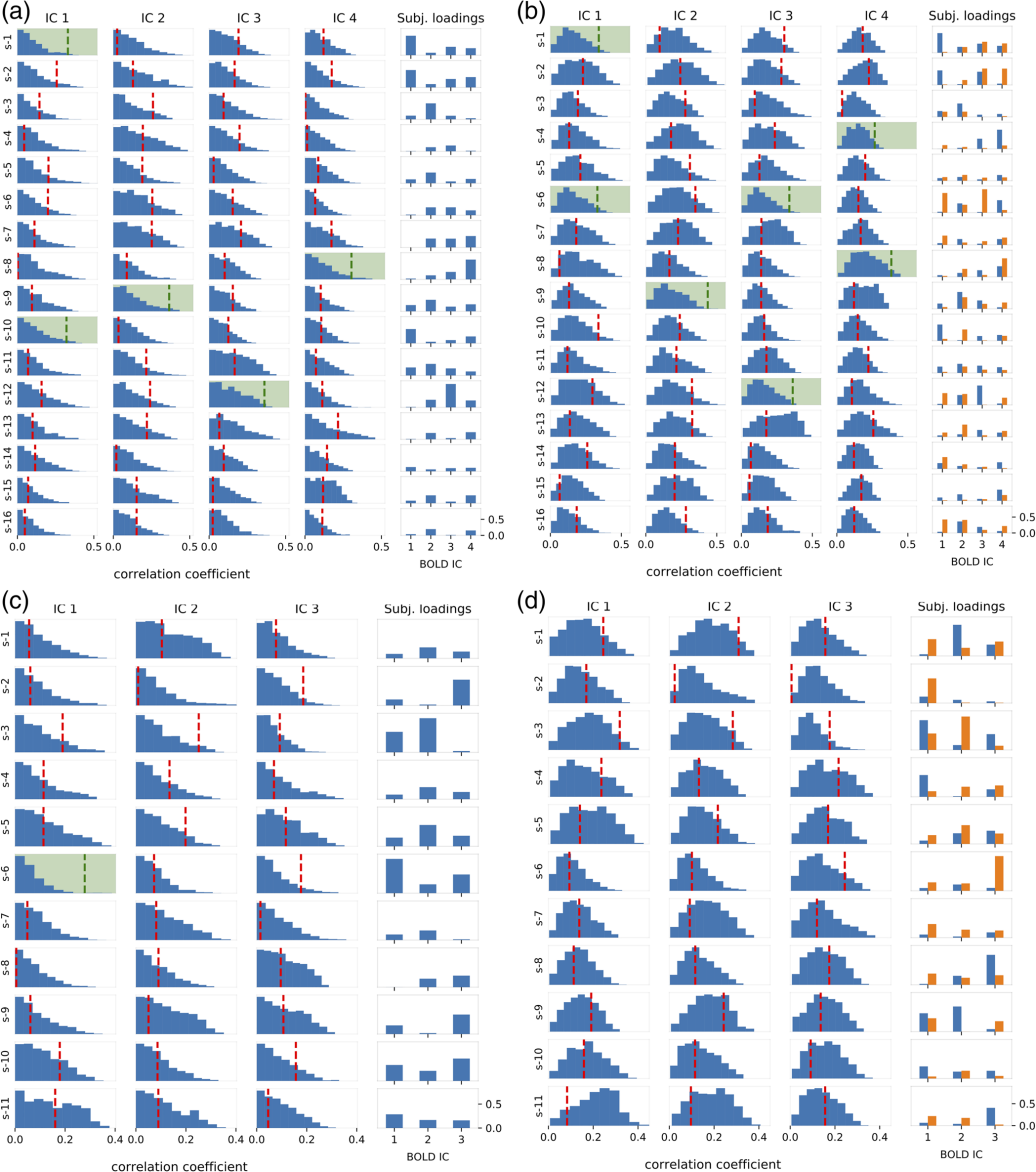
Statistical inference of goodness‐of‐fit based on correlation coefficients. Motor‐imagery data: Panel a for 1 CPD component, panel b for 2 CPD components. Resting‐state data: Panel c for 1 CPD component, panel d for 2 CPD components. For each BOLD IC and subject, correlation coefficient of BOLD estimate (vertical dotted line) is compared to the null distribution (histogram). Dotted line is green if correlation coefficient exceeded the 95th percentile of null distribution, corresponding to a *p* = .05 statistical threshold and red if otherwise. Background is green when the null hypothesis was rejected. The far‐right column of each panel shows absolute values of weightings across subjects and BOLD independent components (ICs). Spatial coverage for motor‐imagery data (i.e. panels a,b): IC1—Left primary motor and somatosensory cortex; IC2—Bilateral primary somatosensory cortex and inferior parietal lobule; IC3—Bilateral primary motor and somatosensory cortex; IC4—Bilateral premotor cortex. Resting‐state networks (i.e. panels c,d): IC1—Default mode network; IC2—Somatosensory network; IC3—Visual network

Histograms of these null distributions across different subjects and ICs are shown in Figure 11 for the motor‐imagery (Figure 11a,b) and resting‐state (Figure 11c,d) datasets, alongside the original correlation coefficients marked by vertical dotted lines. Histograms with a green background signify that the null hypothesis was rejected at the *p* = .05 significance threshold. Also, the vertical dotted line is green when the null hypothesis was rejected and red otherwise.

The right‐most column of each panel of Figure 11 also displays the absolute value of subject weightings for different ICs. This arrangement allows to compare the subject weightings to the pattern of significant correlation coefficients. For motor‐imagery data, we observe from Figure 11a that a subject with a strong weighting for a given IC was more likely to have a correlation coefficient which exceeded the statistical threshold, and vice‐versa. This pattern also holds for Figure 11b where 2 CPD components are used, therefore yielding 2 weightings per subject. Although these observations do not hold for resting‐state data (Figure 11c,d) due to the near‐total absence of significant correlations, it is still the case that stronger subject weightings translate into higher correlations.

As alluded to within the previous paragraph, another important observation is the rate at which the null hypothesis was rejected for motor‐imagery data (Figure 11a,b) compared to resting‐state data (Figure 11c,d). For motor imagery data, the null hypothesis was rejected around 7.8% (5/64) of the time when using 1 CPD component and around 10.9% (7/64) when using 2 CPD components. For resting‐state data, the null hypothesis was rejected 3.03% (1/33) and 0.0% (0/33) of the time.

Moreover, we repeated the process of creating null distributions by bootstrapping BOLD data instead of performing phase randomisation. This proved to be a more liberal way of generating null distributions, resulting in more occurrences where the null hypothesis is rejected. This was done in light of the observed stringency of phase‐randomisation considering the narrowband activity of BOLD‐fMRI data, especially at rest where no stimuli is thought to affect BOLD dynamics, thus keeping the frequency content unaltered and narrowband. The related histograms and results are provided in the Supporting Information in Figure S16 for motor‐imagery and resting‐state data and Figure S17 for supplementary motor‐imagery data.

## 5 DISCUSSION

### 5.1 Simulated data

We proposed a method for estimating region‐specific HRFs while simultaneously deriving from electrophysiology data the spatial and spectral correlates of BOLD data. Our approach was first applied to simulated data, employing a whole‐brain model of coupled modified Stuart–Landau oscillators. We performed phase‐randomisation to perform statistical inference.

We used a basis function expansion technique using the spherical Laguerre functions to obtain robust HRF estimates. This approach has the benefit of requiring less data for estimating impulse response functions than other common approaches, which is advantageous when using data sampled at a low sampling rate such as is the case with experimental BOLD data. Moreover, down‐sampling of EEG data can be performed after convolution with the basis functions, reducing the information loss which accompanies down‐sampling. Nonetheless, such benefits in robustness of HRF estimate come at a cost of imposing bias on the shape of the HRF. Fortunately, selecting the number of spherical Laguerre basis functions may serve to adjust the bias‐variance trade‐off. Hence, the design of our approach is directly well‐suited for empirical data.

Our approach was able to correctly extract the node‐wise frequency bands implemented in the simulations. Specifically, the extracted frequency bands directly reflected the tuning of Stuart‐Landau oscillators at 2 Hz for the right hemisphere and 10 Hz for the left hemisphere (i.e. Figure 3e). Intriguingly, injecting low‐amplitude input noise (e.g. $k_{i}=0.01$) resulted in a better separation of frequency bands compared to the noiseless scenario. Upon further investigation, this was found to be likely due to leakage during passband filtering prior to the Hilbert transform of individual narrowband signals. Specifically, the α amplitude dynamics would leak into the θ and β bands. The θ and β amplitude dynamics thus became highly correlated to α and were thus extracted by the CPD, despite having much lower amplitude than α. On the other hand, for nonzero $k_{i}$, the α dynamics which leaked into the θ and β bands were diluted by input measurement noise. Although some correlation remains between α, θ and β despite the input measurement noise, it was significantly reduced which resulted in a clearer separation of bands.

Moreover, despite our focus on a linear characterisation of the balloon model, it should be noted that the balloon model's nonlinear characteristics have been discussed before, with the form of its second‐order Volterra kernel expansion suggesting an underlying Wiener structure (Friston, Josephs, et al., 1998). In turn, this suggests that the balloon model may be better approximated by a linear dynamic impulse response (i.e. HRF) followed by a static quadratic response. The response of the balloon model thus nonetheless remains mostly linear for low‐amplitude input signals (Friston, Fletcher, et al., 1998). Hence, the estimated HRFs still reflects adequate linear approximations of the balloon model and its parametrisation.

An important goal of this study was to verify whether the derived HRF, spatial distributions and spectral distributions may be used to reconstruct the BOLD signal. We chose the N‐mode product for this reconstruction, although other options are possible. A key assumption in using CPD and the N‐mode product is that the HRF tensor is an adequate low‐rank trilinear representation of the distribution of SSRFs. In an attempt to test this assumption, we performed statistical inference for different noise regimes as shown in Figure 4. As expected, random input and output measurement noise tends to affect the goodness‐of‐fit of BOLD estimates uniformly across nodes. On the other hand, physiological noise expressed through SLFOs (e.g. Figure S2) affect nodes in a more structured manner. This is expected, as our group had previously identified regional variability in the confounding effects of respiratory and cardiac processes (Kassinopoulos & Mitsis, 2019). This regional variability was captured within statistical maps which were used in this current study for deriving the effect of SLFOs on different nodes.

Our approach of applying the CPD onto the HRF tensor is in part inspired by the multiway partial least‐squares (PLS) method employed in Martínez‐Montes et al. (2004) for fusing EEG and BOLD‐fMRI. In this study by Martinez‐Montes and colleagues a third‐order tensor of EEG data was formed by time‐frequency decomposition, akin to the LFP tensor in our work. However, in their study, the time‐series were convolved with the canonical HRF. Multiway PLS was then performed by computing the covariance between the convolved EEG signals and BOLD signals. This resulted in a covariance matrix, which was decomposed using singular value decomposition before reconstructing the data in their original dimensionalities. On the other hand, the HRF tensor in our work may be considered as a correlation tensor rather than a covariance matrix. In other words, it can be viewed as a collection of lag‐based covariance matrices which are concatenated across the temporal dimension to form a third order tensor. This interpretation is based on the view of an impulse response function approximating the cross‐correlation between two signals, corrected for the autocorrelation of the input signal (Westwick & Kearney, 2003). Therefore, we perform the CPD of a correlation tensor rather than the singular value decomposition of a covariance matrix. This distinction allows for the estimation of an HRF following CPD. Another approach similar in spirit to the CPD decomposition of an HRF tensor is the temporal kernel canonical correlation analysis which serves to generalize the cross‐correlogram to high‐dimensional multivariate data as demonstrated in (Biessmann et al., 2010) for fusing neurophysiological recordings with BOLD data.

### 5.2 Empirical data

We applied our method to three different open‐source EEG‐fMRI datasets. Due to the multi‐subject nature of these datasets, HRF tensors were extended to fourth‐order to encapsulate a subject dimension alongside the temporal, spatial and spectral ones. To achieve better computational performance and limit the number of outputs, we performed group‐level data reduction for both EEG and BOLD‐fMRI data. Group ICA of BOLD data followed by dual regression provided group spatial maps and associated subject‐specific time‐series. A similar approach was employed for EEG using SOBI.

The spatial and spectral distributions returned by CPD enabled us to perform a weighted‐averaging of EEG frequency‐band amplitude signals across different SOBI components. Such weighted‐averaging gives rise to what we have termed a compound EEG signal which is convolved with the CPD‐derived HRF to output an estimate of the BOLD IC time‐series. Correlation coefficients were used to assess the goodness‐of‐fit of this BOLD signal estimate. Moreover, delete‐2 jackknifing was employed to assess confidence intervals of the CPD outputs.

Overall, our results suggest that the proposed framework is able to obtain a detailed quantitative representation of EEG‐to‐BOLD neurovascular coupling mechanisms from empirical data. On occasions, such representations allow for an adequate reconstruction of BOLD dynamics using EEG data and data‐driven HRF estimates. The ability to incorporate multiple CPD components provides further insight into different combinations of EEG features which best explain the BOLD signal in a subject‐specific manner. For instance, in several cases, we observed that adding a second CPD component allowed us to decompose broadband frequency distributions into finer sub‐bands. This is an indication that our method can unravel data‐specific frequency bands which may not necessarily align with the standard boundaries of classical EEG frequency bands.

### 5.3 Motor‐imagery dataset

A first observation indicating the robustness of our method is the stability of CPD outputs for the motor‐imagery dataset when 1 CPD component was used (Figure 7a–d), especially for the last two ICs (Figure 7c,d), for which confidence intervals were found to be tightly bound around the median jackknife values. Moreover, these results suggest a broadband relation between distinct EEG frequency bands and the BOLD signal within motor and somatosensory areas for ICs 2–4 (Figure 7b–d), while also exhibiting anti‐correlated activity between visual and motor SOBI components (panels B). On the other hand, the distributions for IC1 (Figure 7a) were sparser and suggest a strong activation at 16 Hz and milder anti‐correlated activations within the upper‐beta range (24–32 Hz). Sparsity was expected as a result of the high 75th percentile soft‐threshold used for IC1 as a result of the parameter sweep (Table 1).

Adding a second CPD component for motor‐imagery data may provide a more complete view of the EEG‐to‐BOLD neurovascular coupling, sometimes however at the cost of higher variability (Figure 8a–d). For instance, the first CPD component of IC1 still exhibited a strong activation at 16 Hz, whereas spectral weightings for the second CPD component are split at 16 Hz between low and high frequencies (Figure 8a‐C). Hence, for subjects with strong weightings for the first component (Figure 8a‐D) upper‐beta activity was anti‐correlated to this pronounced 16 Hz component, whereas subjects with strong weightings for the second component showed correlated activity between upper‐beta and the 4–8 Hz delta range. However, the HRF of the second component exhibited some degree of variability (Figure 8a‐A), with confidence intervals even overlapping with the HRF of the first component, perhaps due to some residual crosstalk between components. Consequently, the spatial (Figure 8a‐B), frequency (Figure 8a‐C) and subject‐level (Figure 8a‐D) distributions also exhibited a larger degree of variability.

In contrast to IC1, where adding a second CPD component comes with a cost of increased variability, adding a second component for IC2 yielded more insightful CPD outputs without comparable shortcomings (Figure 8b). Specifically, both HRFs shown in Figure 8b‐A have tight confidence intervals. Also, despite the similarity in their shapes which could indicate some degeneracy from CPD, the HRF of the second component (orange) exhibits a significantly stronger undershoot than that of the first component (blue). This decoupling in undershoot strength is important considering how the confidence interval for the HRF in Figure 7b‐A—where only 1 CPD was used—grows in magnitude precisely at the undershoot. It thus seems that using two HRFs leads to a more fine‐tuned modelling of BOLD undershoot dynamics. The corresponding spectral distributions (Figure 8b‐C) also bear important insight and therefore mirror the benefit of adding an additional CPD component. Whereas the first component suggests correlated broadband activity over all frequencies, the second component is characterised by an inflection point between 20 Hz and 24 Hz. This latter observation is consistent with previous observations regarding the contribution of electrophysiological frequencies below and above 20 Hz, which respectively yielded correlations of opposite sign to the BOLD signal (Magri et al., 2012; Marecek et al., 2016; Murta et al., 2015). This key observation cannot be drawn from the spectral distribution shown in Figure 8b‐C where only one CPD component was used.

Moreover, comparing correlations between the BOLD signal and its estimate for 1 and 2 CPD components (i.e. Figure 7b‐E vs. Figure 8b‐E) also revealed an increase in the correlation from 0.37 to 0.44 for subject 9 (the subject with the largest correlation in this case). It is likely that the more fine‐tuned modelling of BOLD dynamics and incorporation of anti‐correlated oscillatory activity gives rise to this increased performance. Though the results for IC3 and IC4 became more variable with the added CPD (Figure 8c,d), IC4 also exhibited a non‐negligible increase in correlation from 0.30 to 0.38 for subject 8.

### 5.4 Resting‐state dataset

Similarly to motor‐imagery data, frequency distributions for resting‐state data tended to be relatively broadband; however, some pronounced peaks were also observed within the alpha and low‐gamma bands for DMN (IC1, Figure 9a‐C), upper‐beta for SN (IC2, Figure 9b‐C) and lower/mid‐beta for VN (IC3, Figure 9c‐C). This broadband nature of coupling between resting‐state EEG and RSNs is generally in line with results from (Mantini et al., 2007; Prokopiou, Xifra‐Porxas, et al., 2022).

On the other hand, authors of (Mantini et al., 2007) observe for DMN a peak positive association within the beta‐band which differs from the alpha/low‐gamma association we observed when using 1 CPD component (Figure 9a‐C). Nonetheless, using 2 CPD components (Figure 10a‐C) suggests a possible decoupling between alpha and beta across the second and first components, respectively, suggesting that alpha and beta activity may couple separately to the DMN BOLD signal. Although we considered many different frequencies in our analysis, the alpha‐band is, to the best of our knowledge, the most widely studied rhythm when correlating EEG oscillations to DMN dynamics. Notwithstanding, there are conflicting results across the literature regarding the correlation pattern between alpha and DMN at resting‐state which are discussed in (Bowman et al., 2017).

Comparing our results for the SM RSN (IC2, Figure 9b‐C) with those of (Mantini et al., 2007), we also observe a peak activation in beta with some degree of activation in gamma. Adding a second CPD component results in alpha also yielding contributions (Figure 10b‐C). It is interesting to note that adding a second CPD component stabilised the HRF associated with beta activation. This further indicates that using multiple CPD components may alleviate interference across frequency bands in addition to providing a more complete view of neurovascular coupling mechanisms. In such cases, more CPD components may lead to less variability and tighter confidence intervals.

Results for the VN shows some consistency with previous literature. For instance, when using 1 CPD component (Figure 9c), a strong weighting for EEG SOBI component 3 suggests the involvement of an occipital EEG source in the dynamics of the BOLD signal of VN (Figure 9c‐C). This result is in agreement with prior studies which have observed coupling between occipital EEG oscillations and BOLD activity in visual regions (Goldman et al., 2002; Martínez‐Montes et al., 2004; Scheeringa et al., 2012; Tyvaert et al., 2008). However, our results did not demonstrate a clear relation between the alpha frequency range and VN BOLD activity when either using 1 or 2 CPD components (Figures 9c‐C and 10c‐C). A possible reason for not reproducing the well‐known relation between alpha oscillations and the VN is the variability of the frequency weightings within the alpha range. Whereas the median values of the frequency weightings within alpha are lower than those within beta, the extremities of its confidence intervals suggest a potential role from alpha. Furthermore, another important observation relating to this absent relation between alpha and VN arises by focusing on subject 6. This subject exhibits the strongest correlation coefficient (Figure 10c‐E) despite the median value of its subject weighting for the second component being relatively small. However, the confidence interval of its subject weighting stretches towards stronger weightings (Figure 10c‐D). It is possible that higher median frequency weightings for alpha and a higher subject weighting for subject 6 (amongst other subjects) would go hand‐in‐hand. However, a clear relation between alpha and VN mediated by subject 6 is being masked by the high variability of CPD outputs from which ensues an inability to return strong median weightings. Crosstalk between HRFs (Figure 10c‐A) is also an indication of variability, which may have in turn resulted in the inconsistent correlations obtained between alpha and VN BOLD activity. Finally, inter‐subject variability in EEG‐fMRI correlation patterns (de Munck et al., 2007; Gonçalves et al., 2006) is perhaps in part responsible for this missing relation between alpha oscillations and VN, which warrants a research framework which accounts for this type of variability.

### 5.5 Statistical inference for BOLD estimates

Figure 11 provides an overview of the performance of the proposed approach using CPD outputs and EEG amplitude signals for estimating BOLD data fluctuations. Better BOLD signal estimates were obtained when our method was applied to motor‐imagery data compared to resting‐state data. This is perhaps unsurprising, considering the lower SNR of resting‐state data. Other examples consistent with this finding can be found in previous work from our group where amplitude dynamics of EEG oscillations were also used to model BOLD data. Figure 7 from Prokopiou, Xifra‐Porxas, et al. (2022) and figure 8 from Prokopiou, Kassinopoulos, et al. (2020) both show EEG‐derived estimates of BOLD time‐series for task‐based and resting‐state data. In both cases, more variance of the BOLD signal is captured for task‐based data than resting‐state data.

For motor‐imagery data (Figure 11a,b), adding a second CPD component leads to more statistically significant BOLD signal estimates despite the higher correlation coefficients of the corresponding null distributions. This indicates that overfitting is not the cause of increased performance. This is not the case for resting‐state data (Figure 11c,d), where adding a second CPD components did not provide better BOLD signal estimates. Moreover, for resting‐state data, overfitting is likely considering the large number of EEG SOBI components retained for resting‐state data compared to motor‐imagery data (i.e. 7 vs. 5 components, respectively).

With respect to the relatively low number of significant correlations observed in Figure 11, it is important to note that we explicitly anticipated correlations for some subjects to be insignificant for a given component. This point can be better understood by inspecting the bar charts shown in the right‐most column of each subfigure of Figure 11. Small subject weightings shown in these bar charts would indeed suggest that a significant correlation would be incompatible with the outputs of CPD. Subject 4 in Figure 11a is a good example of this point across all four components. From this example, we could thus conclude that additional CPD components are required in order to allow significant correlations between real and estimated BOLD signals for Subject 4. Moreover, adding a second CPD component for this subject, as shown in Figure 11b, results in stronger subject weightings and a significant correlation for BOLD IC 4. Had this second CPD component been insufficient, as is the case for Subject 14 for instance, adding a third CPD component could be beneficial.

It is also worth noting that the generation of null data by way of phase randomisation translates into quite stringent statistical evaluations. As previously mentioned, we also provide results where phase randomisation was replaced by bootstrapping of the BOLD data, leading to much more lenient conditions for statistical inference. In this situation, we see a net rise in the number of rejected null hypotheses which is accentuated when using 2 CPD components as can be seen in Figures S16 and S17 from the Supplementary Material.

### 5.6 Limitations and potential improvements

Due to our highly multifactorial approach, it is not straightforward to directly compare our results to the previous relevant literature. In general, EEG‐fMRI research is at an early stage where consensus on relations between EEG and BOLD‐fMRI activity is still missing. Much variability in the coupling between EEG and BOLD‐fMRI has been reported as a function of sex, age, individuals and state (Gonçalves et al., 2006; Kumral et al., 2020). This lack of consensus is moreover worsened by the technical challenges related to EEG‐fMRI and the resulting low signal‐to‐noise ratio. Hence, some of our results may not align with expected outcomes, but these expectations are not quite yet grounded on strong consensual views from the literature. Moreover, by accounting for all EEG correlates of BOLD dynamics simultaneously it is not straightforward to claim, as an example, whether beta‐band desynchronisation is correlated with increased BOLD activity in motor and somatosensory areas. As SOBI may return different motor‐related components, these may couple differently to a given task‐related BOLD IC. This was broadly the logic adopted by (Bowman et al., 2017) for explaining discrepancies in relating alpha activity to BOLD DMN activity. In addition, the ability of our method to capture an adequate HRF which reflects the correlation pattern between electrophysiological and BOLD activity comes in stark contrast to assuming a canonical form of the HRF and thus of how EEG frequency‐bands correlate to BOLD dynamics. Moreover, our results suggest that a relationship between beta‐band and BOLD in motor areas holds for certain subjects but does not for others. Results for subject weightings shown in Figures 7a‐D and 8a‐D are good examples of this observation.

In principle, our method provides a more nuanced view of electrophysiology‐driven neurovascular coupling. However, some of the outputs yielded large confidence intervals, suggesting variability of CPD throughout jackknife iterations. The main issue which directly concerns our methodology is the regularisation of the CPD decomposition. The amount of noise in EEG‐fMRI data makes overfitting more likely, especially for high‐dimensional/higher‐order decomposition methods such as CPD. Moreover, complete removal of the gradient artefact and ballistocardiogram artefact is challenging. It is quite possible that residual noise from these artefacts have persisted throughout our analysis. To address these issues, we regularised the CPD decomposition of the HRF tensor using a soft‐thresholding approach. However, other approaches could ensure a more robust CPD decomposition. Standard norm‐based regularisation could sparsify the outputs or further limit overfitting. For instance, a non‐negativity constraint, although not advisable for HRF shape, could limit the variability of jackknife outputs by restricting the sign of weightings to be positive. Non‐negative subject weightings could also render inter‐subject comparisons more interpretable (Cong et al., 2015). The application of different penalty terms for smoothness, sparsity, orthogonality and nonnegativity could be harmonised into a single framework as described in Karahan et al. (2015) within the context of tensor‐based multimodal brain imaging fusion. Furthermore, improvements to our CPD‐based HRF estimation method could allow for a larger number of basis functions, allowing more flexible HRF estimation.

Considering that using 2 CPD components resulted in better BOLD signal estimates compared to using 1 CPD component (e.g. Figure 11a, Subject 4, IC 4), it is also possible that a higher number of components (i.e. >2) is required to adequately represent variability across different dimensions of the HRF tensor. Alternative tensor decompositions which differ from CPD when using multiple components could be considered for deriving electrophysiological features from the HRF tensor. For instance, the Tucker decomposition allows interactions between modes across different components which could limit some of the degenerate outputs as seen between HRFs in Figure 10c‐A.

The variability of CPD outputs is most evident for resting‐state data which is unsurprising considering the low repetition time (2.16 s). A slower TR further compromises the ability to model BOLD dynamics and typically imposes a more aggressive down‐sampling of EEG data. Moreover, EEG data collected in an active MR environment is notoriously noisy and challenging to clean. Poorer EEG SNR further compromises the identification of EEG correlates of BOLD data, especially when using inherently low SNR BOLD resting‐state data. In brief, for either task‐based or resting‐state data, relating EEG features to BOLD‐fMRI is challenging due to the nature of EEG‐fMRI acquisitions. Even for faster TRs used in the literature for whole‐brain acquisitions—for example, 0.72 s for data from the Human Connectome Project—the very low sampling rate of BOLD data in comparison to that of EEG data represents a fundamental limitation to EEG‐fMRI analyses.

A different multimodal arrangement which mitigates these issues is the simultaneous acquisition of EEG with functional near‐infrared spectroscopy (i.e. EEG‐fNIRS; Pellegrino et al., 2016), whereby the sampling frequency of fNIRS is roughly an order of magnitude higher than that of BOLD‐fMRI. Whereas BOLD‐fMRI is mainly sensitive to fluctuations in deoxy‐hemoglobin, fNIRS captures deoxy‐ as well as oxy‐hemoglobin (Machado et al., 2021) providing an additional biological standpoint from which to study neurovascular coupling using EEG as the electrophysiological substrate. It is noteworthy that fNIRS is constrained to more confined and superficial spatial coverage. The use of this modality thus represents a trade‐off between spatial coverage and temporal resolution.

It is also possible that another type of group‐level BSS algorithm may be better suited for decomposing EEG data into components. We used SOBI in part due to its relative robustness against inter‐subject variability in the underlying mixing matrices (Lio & Boulinguez, 2013), although this advantage over higher‐order based methods has not been found to be ubiquitous (Huster et al., 2015). Furthermore, algorithms based on second‐order statistics such as SOBI depend (although not always directly) on the frequency content of the data for ensuring that the derived components are orthogonal. Hence, it may seem counter‐intuitive to decompose EEG data into orthogonal components based on their frequency contents to then attempt to capture collinearity between SOBI components via CPD. However, collinearity between SOBI components is mediated through their respective SSRFs rather than SOBI time‐series from which orthogonality is imposed. Moreover, SOBI time‐series were subjected to multi‐taper filtering to obtain the output amplitude signals. It is these amplitude signals rather than the original SOBI time‐series which were used to estimate SSRFs, rendering it possible for SSRFs to correlate across frequencies.

## 6 CONCLUSION

The proposed methodology enables us to extract spatial and spectral features derived from electrophysiology while simultaneously estimating HRFs on a node‐by‐node or components basis. Our approach was first tested on simulated LFP and BOLD data and involved the implementation of a novel variant of Stuart‐Landau oscillators to generate LFP oscillatory amplitude dynamics. Then, open‐source EEG‐fMRI data was used to further validate our method.

Our results suggest that, when applied to simulated data, our methodology can accurately extract node‐wise spatial and spectral distributions as well as node‐specific HRFs. We tested the performance of our method under different noise regimes, involving input and output measurement noise as well as physiological confounds. Results are in agreement with the whole‐brain modelling parametrisation; for instance, the frequency bands extracted across nodes matched the natural frequencies of the fast SLO subpopulations that we set for simulating data. We also show that for moderate amounts of noise introduced into the data, our method is capable of reconstructing BOLD data using correlations as a goodness‐of‐fit metric. However, noise regimes with heavier noise contributions result in an inability to model BOLD signals.

To test our method on empirical EEG‐fMRI data, we leveraged CPD applied to fourth‐order HRF tensors to investigate EEG‐to‐BOLD neurovascular coupling, as well as its inter‐subject variability. Our method suggests that broadband distributions of frequencies mediate this type of neurovascular coupling, especially for motor‐imagery data. For both motor‐imagery and resting‐state datasets, broadband frequency distributions may be further decomposed into sub‐bands by adding a second component to the CPD decomposition. Moreover, CPD outputs can be used to reconstruct adequate BOLD signal estimates.

This article represents a proof‐of‐concept of employing a tensor decomposition framework for studying subject‐specific neurovascular coupling mechanisms in a more general context. It allows the simultaneous incorporation of broadband electrophysiological activity combined with flexibility in the HRF dynamics with respect to both electrophysiological frequency and spatial location. Potential improvements to our approach include regularisation, exploring a larger number of CPD components and alternative tensor decomposition algorithms.

## FUNDING INFORMATION

This work was supported by the Natural Sciences and Engineering Research Council of Canada (Discovery Grant 06638‐2019 awarded to Georgios D. Mitsis), the Fonds de recherche du Québec—Nature et technologies (FRQNT; Team Grant PR254860 awarded to Georgios D. Mitsis) and the Canada First Research Excellence Fund (awarded to McGill University for the Healthy Brains for Healthy Lives initiative).

## CONFLICT OF INTEREST

The authors declare no conflicts of interest.

## PERMISSION TO REPRODUCE MATERIAL FROM OTHER SOURCES

Permission was granted by Dr Joana Cabral to adapt figure 2 of Cabral et al. (2014) for the purpose of designing Figure 1 of the present manuscript.

## Supporting information


**FIGURE S1**: Simulated LFP and BOLD signals. LFP signals were simulated using modified Stuart‐Landau oscillators, consisting of a slow subpopulation (a) and a fast subpopulation (b). The squared time‐course of the fast subpopulation served as the driving signal to the balloon model, giving rise to the BOLD signal (c).
**FIGURE S2**: Simulated physiological signals and SLFOs. The simulated PPG signal (a) was used to obtain the heart‐rate signal (c). The latter was convolved with the cardiac response function (e) to generate the cardiac SLFO (g, red). The respiratory time‐course (b) gave rise to the respiratory flow signal (d). The latter was convolved with the respiratory response function (f) to output the respiratory SLFO (g, blue). Cardiac and respiratory SLFOs were summed to create the SLFO signal (g, green) which was later added to the BOLD signal as a source of physiological confound.
**FIGURE S3**: Canonical polyadic decomposition (CPD). A third order tensor 𝓧 decomposed into a sum of n outer products between factors 𝒂_𝒊_, 𝒃_𝒊_ and 𝒄_𝒊_.
**FIGURE S4**: Spherical Laguerre basis functions, plotted for first three basis functions. Examples when decay rate parameter ***α*** = 0.2 (top), ***α*** = 0.4 (middle) and ***α*** = 0.6 (bottom)
**FIGURE S5**: Scaling of HRF tensor. (a) Individual signals from the LFP tensor are convolved with their respective SSRFs stored within the HRF tensor. This gives rise to a tensor of bold signals. (b) Individual BOLD signals from this latter tensor are correlated with the real BOLD signal. Correlations are stored within a correlation matrix. (c) Soft‐thresholding of correlation matrix. (d) Unweighted HRF tensor scaled by entries of the soft‐thresholded correlation matrix.
**FIGURE S6**: Input and output measurement noise and physiological confounds. Frequency‐band specific input measurement noise were scaled by 𝒌_𝒊_ and added onto LFP signals to produce noisy LFP signals. Output measurement noise was scaled by 𝒌_𝒐_ and SLFO scaled by 𝒌_𝒑_, both added to the BOLD signal to produce a noisy BOLD signal. Noisy LFP and BOLD signals were used to construct the HRF tensor to then finally proceed with CPD
**FIGURE S7**: Residuals‐based block‐bootstrapping. Residuals were derived by subtracting the BOLD signal estimate from the BOLD signal. Residuals were segmented into blocks which were resampled with replacement to form bootstrap replicates of the residuals. Individual bootstrap replicates were added back to the BOLD signal to construct a new HRF tensor and perform CPD for each replicate
**FIGURE S8**: Statistical inference based on phase randomisation of the BOLD signal. Pearson correlations were used to quantify the goodness‐of‐fit of the BOLD signal estimate. The correlation between BOLD signal and BOLD signal estimate (purple) was compared against a null distribution of correlations (orange). This null distribution is constructed by phase‐randomising the BOLD signal numerous times. For each phase randomised BOLD signal, an estimate of the phase randomised BOLD signal was derived. Every pair of phase‐randomised BOLD signal and its estimate were correlated and added to the null distribution.
**FIGURE S9**: Two different 64‐channel EEG electrode layouts used for the resting‐state dataset, when loaded in EEGLAB. Eleven subjects were recorded using the layout shown in panel a whereas five subjects were recorded using the layout shown in panel b. Only subjects recorded with layout a were used for our analysis.
**FIGURE S10**: Results for simulated data in right caudal anterior‐cingulate cortex. (a) Location of node of interest, results for which are shown in other subfigures. (b) Estimated spatial distribution. (c) Proxy of ground‐truth for spatial distribution (i.e. reference distribution) obtained by correlating signals of slow subpopulations across nodes. (d) Estimated and reference HRFs. Refer to SM 3 for methodology related to reference HRFs. Refer to Figure d2 for a comparison between estimated and reference HRFs where the post‐stimulus undershoot is absent. (e) Spectral distribution. Confidence intervals for panels d and e obtained using methodology described in SM 4. FIGURE S10d2: Modified replicate of Figure d, where the autoregulation parameter of the balloon model has been increased during simulation. This increase in parameter value results in the absence of a post‐stimulus undershoot, as can be observed in this figure. Comparison between estimated HRF with CPD using 3 Laguerre functions and reference HRFs. HRF: Hemodynamic Response Function, CPD: Canonical Polyadic Decomposition, OLS: Ordinary Least Squares.
**FIGURE S11**: Comparing the modelling capacity of a CPD‐based HRF with that of the canonical HRF. BOLD signals were simulated for all 66 nodes of the cortical parcellation and used to extract spatial and spectral features as well as HRF estimated using our proposed CPD‐based method. The simulated BOLD signals were then either estimated using all CPD outputs or by replacing the CPD‐based HRF by the canonical HRF. For the BOLD signal simulations, the balloon model parameters were selected such that the resulting CPD‐based HRF would differ from the canonical HRF to some extent. Specifically, the signal decay parameter of the balloon model was reduced from 1.54 s to 0.54 s. (a) HRF derived using the CPD decomposition for randomly selected cortical region. (b) Canonical HRF. (c) Simulated BOLD signal (blue) overlaid with BOLD signal estimate (orange) when using CPD‐based HRF for same cortical region as in (a). (d) Simulated BOLD signal (blue) overlaid with BOLD signal estimate (orange) when using canonical HRF for same cortical region as in (a). (e) Histogram of correlations when using CPD‐based HRF for all 66 nodes of the cortical parcellation. (f) Histogram of correlations when using canonical HRF for all 66 nodes of the cortical parcellation. corr: correlation.
**FIGURE S12**: BOLD independent components (IC) in panel a and scalp topography of EEG SOBI components in panel b for supplementary motor‐imagery data. Main spatial coverage for BOLD IC1 is bilateral primary motor, primary somatosensory & premotor; for IC2 is left primary & secondary somatosensory cortex; for IC3 is right secondary somatosensory cortex & inferior parietal lobule; for IC4 is bilateral inferior & superior parietal lobules. Regions of BOLD ICs determined using Juëlich histological atlas.
**FIGURE S13**: Results for supplementary motor‐imagery data when using 1 CPD component. Figures S13.1–S13.4 show results for different BOLD IC (Figure S12a). For each IC, estimated HRF shown in panel a, EEG SOBI topographies (Figure S12b) and spatial distribution in panel b, frequency distribution in panel c, subject distribution in panel D and BOLD signal estimate (orange) alongside real bold signal (blue) in panel e. For panel e, results presented for subject with highest correlation coefficient (r) between BOLD signal estimate and real BOLD signal. The vertical axis of each subfigure bears arbitrary units.
**FIGURE S14**: Results for supplementary motor‐imagery data when using 2 CPD components. Figures S14.1–S14.4 show results for different BOLD IC (Figure S12a). For each IC, estimated HRF shown in panel a, EEG SOBI topographies (Figure S12b) and spatial distribution in panel b, frequency distribution in panel c, subject distribution in panel d and BOLD signal estimate (orange) alongside real BOLD signal (blue) in panel e. For panel e, results presented for subject with highest correlation coefficient (r) between BOLD signal estimate and real BOLD signal. The vertical axis of each subfigure bears arbitrary units.
**FIGURE S15**: Statistical inference of goodness‐of‐fit based on correlation coefficients for supplementary motor data. Panel a for 1 CPD component, panel b for 2 CPD components. For each BOLD IC and subject, correlation coefficient of BOLD estimate (vertical dotted line) is compared to null distribution (histogram). Dotted line is green if correlation coefficient exceeds 95th percentile of null distribution, corresponding to a *p* = .05 statistical threshold and dotted line is red if otherwise. Background is green when null hypothesis rejected. Farright column of each panel shows absolute values of weightings for given subject across ICs. Main spatial coverage for BOLD IC1 is bilateral primary motor, primary somatosensory & premotor cortex; for IC2 is left primary & secondary somatosensory cortex; for IC3 is right secondary somatosensory cortex & inferior parietal lobule; for IC4 is bilateral inferior & superior parietal lobules.
**FIGURE S16**: Statistical inference of goodness‐of‐fit based on correlation coefficients when replacing phase‐randomisation by bootstrapping. Motor‐imagery data: panel a for 1 CPD component, panel b for 2 CPD components. Resting‐state data: panel c for 1 CPD component, panel d for 2 CPD components. For each bold IC and subject, correlation coefficient of BOLD estimate (vertical dotted line) is compared to the null distribution (histogram). Dotted line is green if the correlation coefficient exceeded the 95th percentile of null distribution, corresponding to a *p* = .05 statistical threshold and red if otherwise. Background is green when the null hypothesis was rejected. The far‐right column of each panel shows absolute values of weightings across subjects and BOLD independent components (ICs). Spatial coverage for motor‐imagery data (i.e. Panels a,b): IC1—left primary motor & somatosensory cortex; IC2—bilateral primary somatosensory cortex & inferior parietal lobule; IC3—bilateral primary motor and somatosensory cortex; IC4—bilateral premotor cortex. Resting‐state networks (i.e. Panels c,d): IC1—default mode network; IC2—somatosensory network; IC3—visual network
**FIGURE S17**: Statistical inference of goodness‐of‐fit based on correlation coefficients when replacing phase‐randomisation by bootstrapping. Results shown for supplementary motor‐imagery data. Panel a for 1 CPD component, panel b for 2 CPD components. For each BOLD IC and subject, correlation coefficient of BOLD estimate (vertical dotted line) is compared to null distribution (histogram). Dotted line is green if correlation coefficient exceeds 95th percentile of null distribution, corresponding to a *p* = .05 statistical threshold and dotted line is red if otherwise. Background is green when null hypothesis rejected. Far‐right column of each panel shows absolute values of weightings for given subject across ICs. Main spatial coverage for BOLD IC1 is bilateral primary motor, primary somatosensory & premotor cortex; for IC2 is left primary & secondary somatosensory cortex; for IC3 is right secondary somatosensory cortex & inferior parietal lobule; for IC4 is bilateral inferior & superior parietal lobules

**TABLE S1** Decay‐rate parameter and percentile selected by the parameter sweep for each independent component (IC) of supplementary motor‐imagery dataset. Values shown when number of canonical polyadic decomposition (CPD) components set to 1 and 2.

## Data Availability

Experimental resting‐state data can be accessed at https://osf.io/dvmrb/ in BIDS format. Subjects used for this study are sub‐32, 36, 37, 38, 39, 40, 43, 44, 45, 48, and 49. Pre‐processed EEG data are located in subjects' *eeg* subdirectories within the *derivatives* directory. Readers may refer to (Deligianni et al., 2014; Deligianni et al., 2016) for additional information regarding the resting‐state dataset. The experimental motor‐imagery dataset analysed within the main body of the article can be accessed at 10.18112/openneuro.ds002338.v2.0.0. Data from *run3* were used for analysis. Subject sub‐xp219 was excluded as this subject did not contain BOLD‐fMRI data for *run3*. The experimental motor‐imagery dataset analysed within Supplementary Materials can be accessed at 10.18112/openneuro.ds002336.v2.0.0. For both motor‐imagery datasets, pre‐processed EEG data are located in subjects' *eeg_pp* subdirectories within the *derivatives* directory. Readers may refer to (Lioi et al., 2020) for additional information regarding the motor‐imagery datasets. For all datasets, raw BOLD‐fMRI data were taken from subjects' main directory (i.e. not from the *derivatives* directory).

## Footnotes

^1^
SLFO (systemic low‐frequency oscillation) not to be mistaken with SLO (Stuart–Landau oscillator).

^2^

https://github.com/miykael/atlasreader/blob/master/atlasreader/data/atlases/atlas_desikan_killiany.nii.gz.

^3^
Another term for CPD, used in *Tensorly*, is Parallel Factor Analysis abbreviated as PARAFAC.
