# Supplementary material for: Extracting electrophysiological correlates of functional magnetic resonance imaging data using the canonical polyadic decomposition
Source: Hum Brain Mapp. 2022 May 14;43(13):4045–73. doi: 10.1002/hbm.25902 (PMC9374895; doi:10.1002/hbm.25902)
Supplement: Supplementary file 1 — FIGURE S1: Simulated LFP and BOLD signals. LFP signals were simulated using modified Stuart‐Landau oscillators, consisting of a slow subpopulation (a) and a fast subpopulation (b). The squared time‐course of the fast subpopulation served as the driving signal to the balloon model, giving rise to the BOLD signal (c). FIGURE S2: Simulated physiological signals and SLFOs. The simulated PPG signal (a) was used to obtain the heart‐rate signal (c). The latter was convolved with the cardiac response function (e) to generate the cardiac SLFO (g, red). The respiratory time‐course (b) gave rise to the respiratory flow signal (d). The latter was convolved with the respiratory response function (f) to output the respiratory SLFO (g, blue). Cardiac and respiratory SLFOs were summed to create the SLFO signal (g, green) which was later added to the BOLD signal as a source of physiological confound. FIGURE S3: Canonical polyadic decomposition (CPD). A third order tensor 𝓧 decomposed into a sum of n outer products between factors 𝒂𝒊, 𝒃𝒊 and 𝒄𝒊. FIGURE S4: Spherical Laguerre basis functions, plotted for first three basis functions. Examples when decay rate parameter α = 0.2 (top), α = 0.4 (middle) and α = 0.6 (bottom) FIGURE S5: Scaling of HRF tensor. (a) Individual signals from the LFP tensor are convolved with their respective SSRFs stored within the HRF tensor. This gives rise to a tensor of bold signals. (b) Individual BOLD signals from this latter tensor are correlated with the real BOLD signal. Correlations are stored within a correlation matrix. (c) Soft‐thresholding of correlation matrix. (d) Unweighted HRF tensor scaled by entries of the soft‐thresholded correlation matrix. FIGURE S6: Input and output measurement noise and physiological confounds. Frequency‐band specific input measurement noise were scaled by 𝒌𝒊 and added onto LFP signals to produce noisy LFP signals. Output measurement noise was scaled by 𝒌𝒐 and SLFO scaled by 𝒌𝒑, both added to the BOLD signal to produce a [file HBM-43-4045-s002.pdf]

Supporting Information

Supplementary Methods (SM)

SM 1 – rescaling the HRF tensor using soft-thresholding

As the amplitude of individual SSRFs determines the outputs of CPD, SSRF amplitudes were rescaled to capture the portion of neurovascular coupling which can adequately model the BOLD signal. Figure S.5 shows the steps involved in this rescaling. As shown below in Figure S.5A, the LFP signal from each space & frequency pair was first convolved with its corresponding SSRF to generate a BOLD signal estimate. The estimated output was then correlated to the BOLD signal as a measure of goodness-of-fit, which resulted in a matrix of Pearson correlation coefficients (Fig. S.5B), housing one entry for each space-frequency pair. Then, we derived from this matrix the correlation belonging to the 95th percentile. This 95th percentile correlation value was used to perform soft-thresholding on the correlations matrix such that correlation values were rescaled (Fig. S.5C). Individual SSRFs were then multiplied by their associated soft-thresholded correlation value (Fig. S.5D). Equation (S.1) shows how the soft-threshold operator S_λ functions. If the magnitude of the input $|x|$ is greater than the threshold λ, then $|x|$ is reduced by λ and the original sign of x is conserved. Otherwise, if $|x|$ falls below λ the output is simply 0. We used the soft-thresholding operator with correlation coefficients as inputs x and the 95th percentile correlation coefficient as threshold λ.

$$S_\lambda(x) = \max(\,|x| - \lambda\,, 0\,) * \operatorname{sgn}(x) \qquad \text{(S.1)}$$

We used soft-thresholding to sparsify the HRF tensor, such that SSRFs associated with correlations below the 95th percentile were nulled. Soft-thresholding - as opposed to hard-thresholding - downscales all correlation coefficients regardless of whether they survive the threshold. To clarify this point, let's consider a threshold of 0.25 and correlation coefficients of 0.5 and 1.0. With hard-thresholding, both values would remain intact, i.e. 0.5 and 1.0. With soft-thresholding however, values are reduced by 0.25, and thus become 0.25 and 0.75. In this case, the ratio between thresholded correlation coefficients goes from 1.0/0.5 = 2 for hard-thresholding to 0.75/0.25 = 3 for soft-thresholding. Soft-thresholding thus has the effect of increasing higher correlations relative to lower correlations, thereby emphasizing differences between correlation coefficients. Consequently, the ratio between SSRF amplitudes are also increased for SSRFs associated with higher correlation coefficients. The intended effect of reweighting the HRF tensor in the way just described was to render CPD more sensitive to SSRFs leading to higher correlations. Moreover, the aforementioned increase in ratio of SSRFs served to sparsify CPD outputs. For clarification, readers may refer to (Hervás et al., 2019) for an explanation of the soft-thresholding operator as the solution to an L1-regularized least-squares regression problem and its application to multiway partial least-squares.

SM 2 – injecting input noise

The simplest way for accounting for the effect of the Hilbert transform on thermal noise is to inject an additive white Gaussian noise (AWGN) process into the data at an early stage of data processing (before the Hilbert transform). A new AWGN process could be injected for each bootstrap or phase randomization iteration, applying the preprocessing steps every time. However, this approach comes at a great computational cost and memory demand during the bootstrapping and construction of empirical distributions for statistical inference. Instead, we used the following mathematical relationship which relates the amplitude of a Hilbert transformed sum of complex signals $z = u_1 + u_2$ (Eq. S.2):

$$|z|^2 = |u_1|^2 + |u_2|^2 + 2(x_1x_2 + y_1y_2) \tag{S.2}$$

where x_1 and y_1 are the real and imaginary parts of u_1 respectively, and x_2 and y_2 are the real and imaginary parts of u_2. The magnitude of a given complex signal is symbolized as $|\cdot|$. Similarly, if we consider u_1 as the Hilbert transformed LFP data and u_2 the Hilbert transformed AWGN process with scaling factor k_i such that $u_2 = k_i(x_2 + iy_2)$, the following relation holds (Eq. S.3):

$$|z|^2 = |u_1|^2 + {k_i}^2|u_2|^2 + k_i[2(x_1x_2 + y_1y_2)] \tag{S.3}$$

According to Equation (S.3), one may compute the squared noise amplitude $|u_2|^2$ and cross-term $2(x_1x_2 + y_1y_2)$ and scale with k_i accordingly. Importantly, rather than computing these two terms at every bootstrap or phase randomisation iteration, the terms $|u_2|^2$ and $2(x_1x_2 + y_1y_2)$ were computed once and replicated by random block resampling at every iteration. In short, the idea is to mimic the process of injecting input measurement noise to the LFP data – with a different AWGN process at every iteration - and performing the Hilbert transform afterwards.

SM 3 – estimating reference hemodynamic response functions

We also obtained reference hemodynamic response functions (HRFs) that served as standards to assess the performance of the proposed HRF estimation scheme. To create these reference HRFs, WGN was directly used as input to the balloon model as opposed to squared LFP signals. Using WGN ensures that the full bandwidth of the system is excited by serially uncorrelated data (Marmarelis & Marmarelis, 1978), serving as an ideal signal to derive a reference HRF. Two reference HRFs were obtained using two separate techniques, the first being the spherical Laguerre basis function expansion technique explained above, and the second technique being the ordinary least-squares method (Westwick & Kearney, 2003). Whereas the first reference HRF reflects the ability of spherical Laguerre basis functions to estimate proper HRFs, the ordinary least-squares method serves in principle as a more accurate estimate of the underlying HRF although potentially less stable. It should be noted that, whereas the number of spherical Laguerre basis functions was set to 3 during the main analysis to avoid

overfitting, the number of basis functions when using WGN was set to 5. In this latter case, we did not observe overfitting despite the larger number of basis functions. This assessment was based on qualitatively observing the derived HRF and looking for non-smooth features within the HRF waveform.

Figures S.10D and S.10D.2 show comparisons between HRFs estimated using CPD and reference HRFs. In Figure S.10D, the balloon model parameters were set such that a post-stimulus undershoot is present in the HRFs. On the other hand, parameters were set in Figure S.10D.2 such that the undershoot is absent. Removing this undershoot was achieved by increasing the autoregulation parameter of the balloon model.

SM 4 – residuals-based block bootstrapping

To assess the robustness of CPD and obtain confidence intervals for the estimated HRFs and spatial-spectral distributions, a residuals-based bootstrapping procedure was performed. As previously mentioned, the HRF and spatial-spectral distributions derived from the CPD decomposition of the HRF tensor were used to obtain a weighted linear combination of LFP signals, yielding a compound LFP signal which was convolved with the estimated HRF. The resulting BOLD signal estimate was compared to the true BOLD signal to obtain the residuals (Fig. 1D-E, Fig. S.7).

Bootstrapping was employed to obtain confidence intervals on the estimated HRFs and spatial-spectral distributions (Fig. S.7). Specifically, the residuals between the estimated BOLD signal and real BOLD signal were randomly re-sampled with replacement. The residuals were resampled in blocks rather than individual datapoints, in light of the serially correlated nature of the residuals. Block resampling was accomplished using the Recombinator Python package by way of the Tapered Block Bootstrap to ensure smooth transitions between resampled data segments. The size of the block was set to the pre-defined length of the HRF.

References

Hervás, D., Prats-Montalbán, J. M., García-Cañaveras, J. C., Lahoz, A., & Ferrer, A. (2019). Sparse N-way partial least squares by L1-penalization. *Chemometrics and Intelligent Laboratory Systems*, *185*(January), 85–91. https://doi.org/10.1016/j.chemolab.2019.01.004

Marmarelis, P. Z., & Marmarelis, V. Z. (1978). The White-Noise Method in System Identification. In *Analysis of Physiological Systems* (pp. 131–180). Springer, Boston, MA.

Westwick, D. T., & Kearney, R. E. (2003). *Identification of Nonlinear Physiological Systems*. John Wiley & Sons, Inc. https://doi.org/10.1002/0471722960

Supplementary Figures:

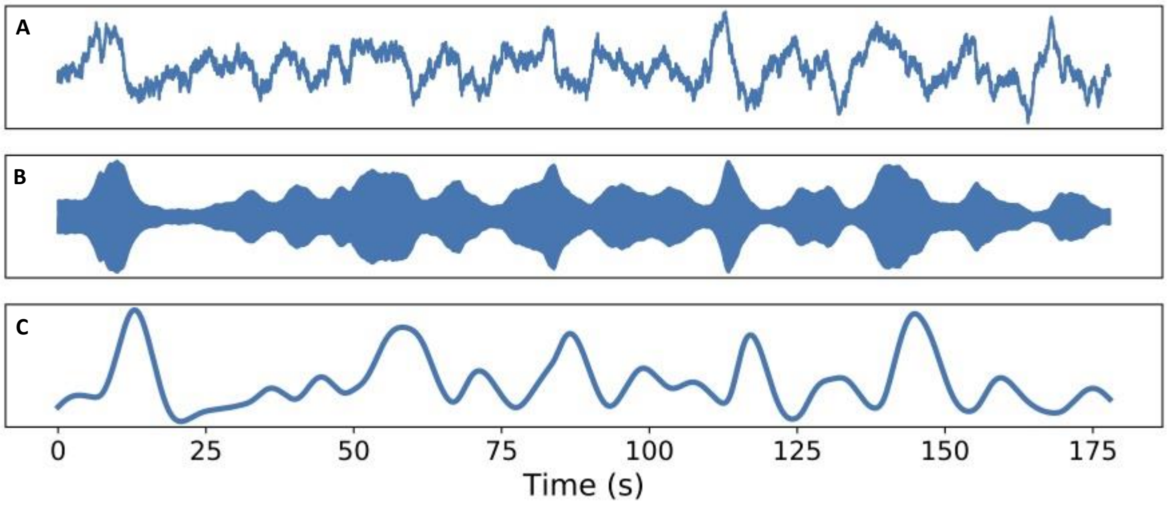

Figure S.1 : Simulated LFP and BOLD signals. LFP signals were simulated using modified Stuart-Landau oscillators, consisting of a slow subpopulation (A) and a fast subpopulation (B). The squared time-course of the fast subpopulation served as the driving signal to the balloon model, giving rise to the BOLD signal (C).

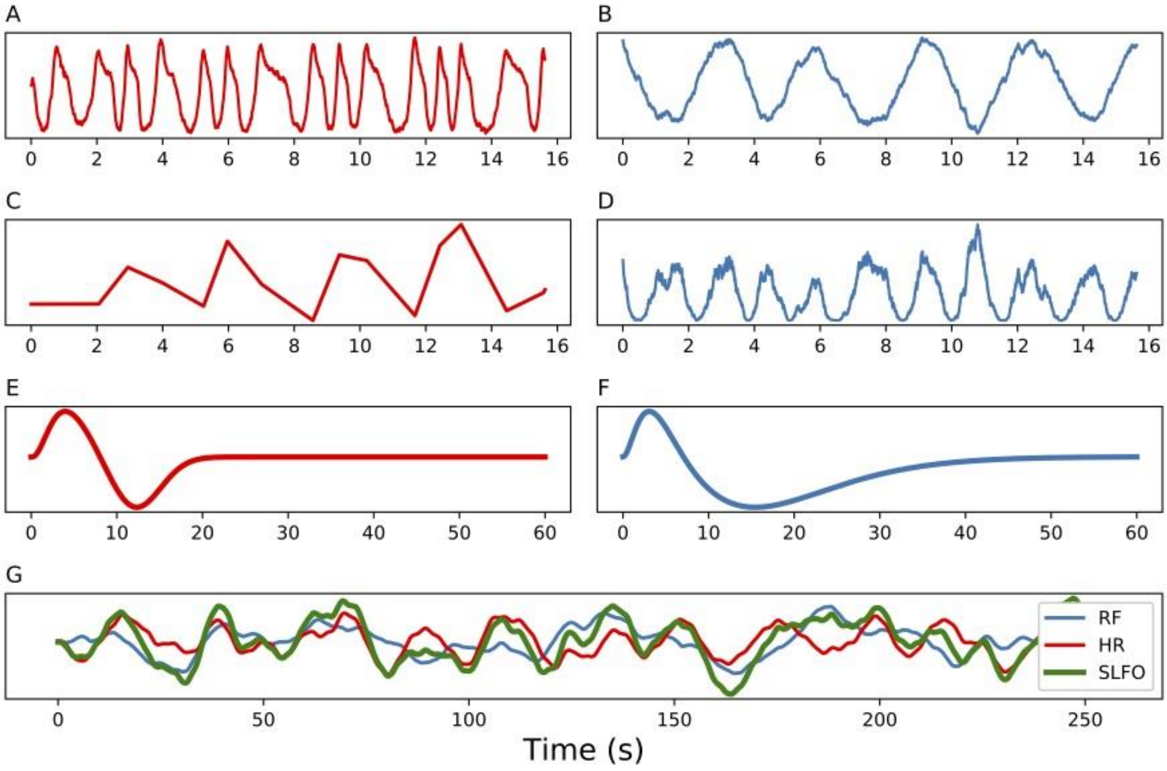

Figure S.2 : Simulated physiological signals and SLFOs. The simulated PPG signal (A) was used to obtain the heart-rate (HR) signal shown in (C). The latter was convolved with the cardiac response function (E) to generate the cardiac SLFO (G, red). The respiratory time-course (B) gave rise to the respiratory flow (RF) signal shown in (D). The latter was convolved with the respiratory response function (F) to output the respiratory SLFO (G, blue). Cardiac and respiratory SLFOs were summed to create the SLFO signal (G, green) which was later added to the BOLD signal as a source of physiological confound.

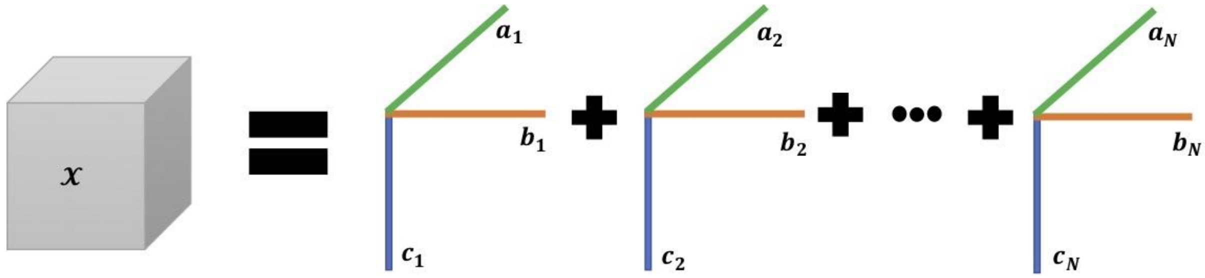

Figure S.3: Canonical polyadic decomposition (CPD). A 3rd order tensor $\boldsymbol{\mathcal{X}}$ decomposed into a sum of *n* outer products between factors $\boldsymbol{a_i}$,$\boldsymbol{b_i}$ and $\boldsymbol{c_i}$.

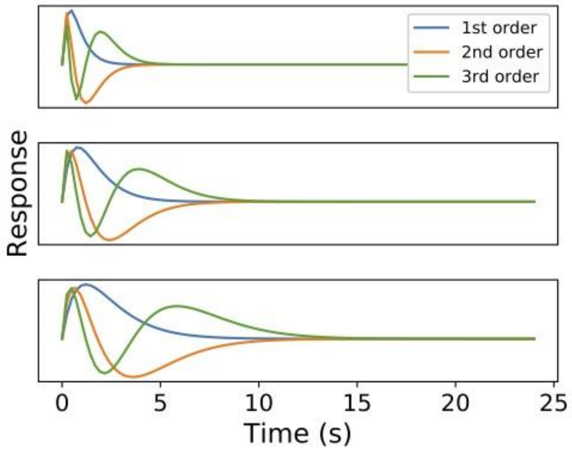

Figure S.4 : Spherical Laguerre basis functions, plotted for first three basis functions. Examples when decay rate parameter $\boldsymbol{\alpha} = \mathbf{0.2}$ (top), $\boldsymbol{\alpha} = \mathbf{0.4}$ (middle) and $\boldsymbol{\alpha} = \mathbf{0.6}$ (bottom)

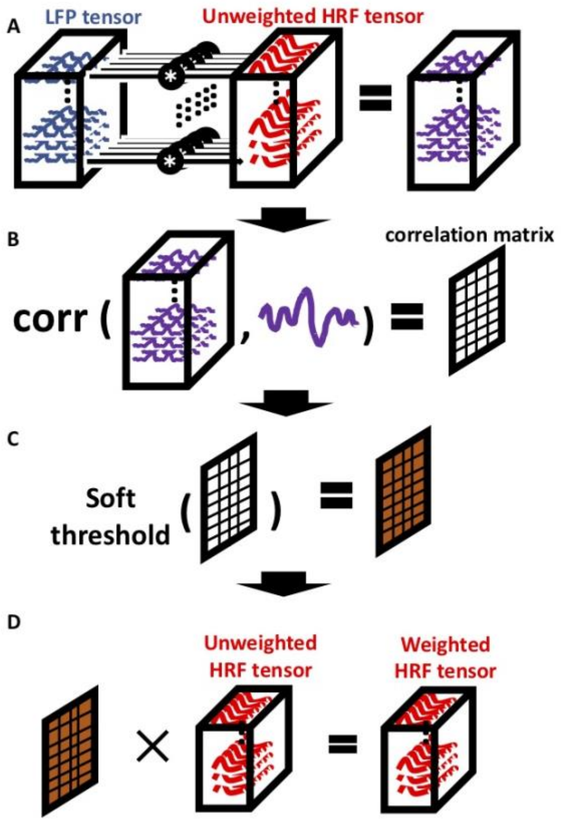

Figure S.5 : Scaling of HRF tensor. A) Individual signals from the LFP tensor are convolved with their respective SSRFs stored within the HRF tensor. These convolutions give rise to a tensor of BOLD signals. B) Correlations are computed between individual BOLD signals from this latter tensor and the real BOLD signal. Correlations are stored within a correlation matrix. C) Soft-thresholding of correlation matrix. D) Unweighted HRF tensor scaled by entries of the soft-thresholded correlation matrix.

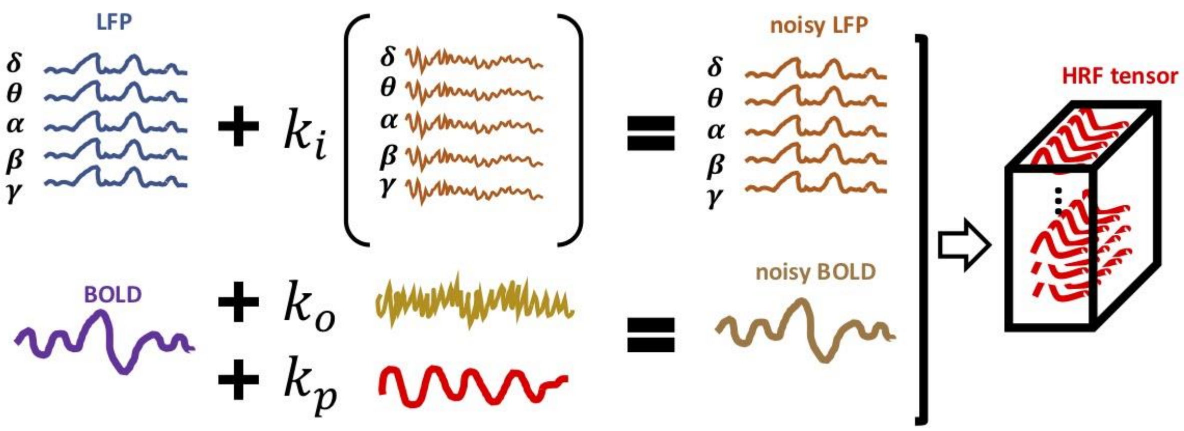

Figure S.6: Input & output measurement noise and physiological confounds. Frequency-band specific input measurement noise were scaled by $\boldsymbol{k_i}$ and added onto LFP signals to produce noisy LFP signals. Output measurement noise was scaled by $\boldsymbol{k_o}$ and SLFO scaled by $\boldsymbol{k_p}$, both added to the BOLD signal to produce a noisy BOLD signal. Noisy LFP and BOLD signals were used to construct the HRF tensor to then finally proceed with CPD.

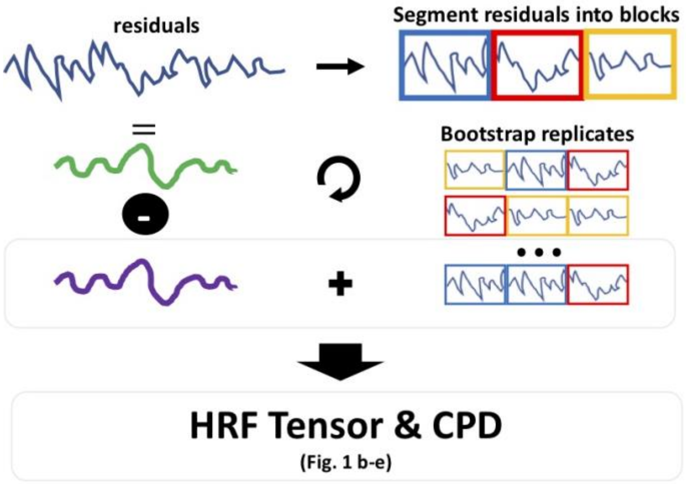

Figure S.7: Residuals-based block-bootstrapping. Residuals were derived by subtracting the BOLD signal estimate from the BOLD signal. Residuals were segmented into blocks which were resampled with replacement to form bootstrap replicates of the residuals. Individual bootstrap replicates were added back to the BOLD signal to construct a new HRF tensor and perform CPD for each replicate.

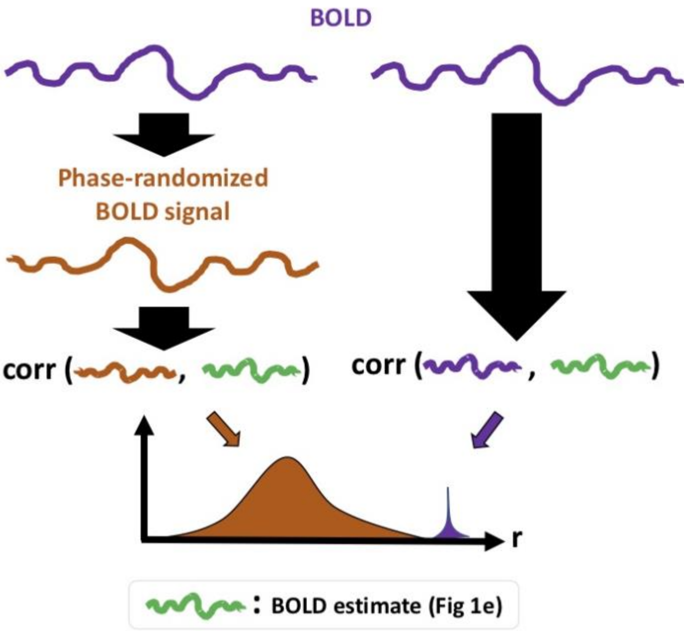

Figure S.8: Statistical inference based on phase-randomisation of the BOLD signal. Pearson correlations were used to quantify the goodness-of-fit of the BOLD signal estimate. The correlation between BOLD signal and BOLD signal estimate (purple) was compared against a null distribution of correlations (orange). This null distribution is constructed by phase-randomising the BOLD signal numerous times. For each phase-randomised BOLD signal, an estimate of the phase-randomised BOLD signal was derived. Every pair of phase-randomised BOLD signal and its estimate were correlated and added to the null distribution.

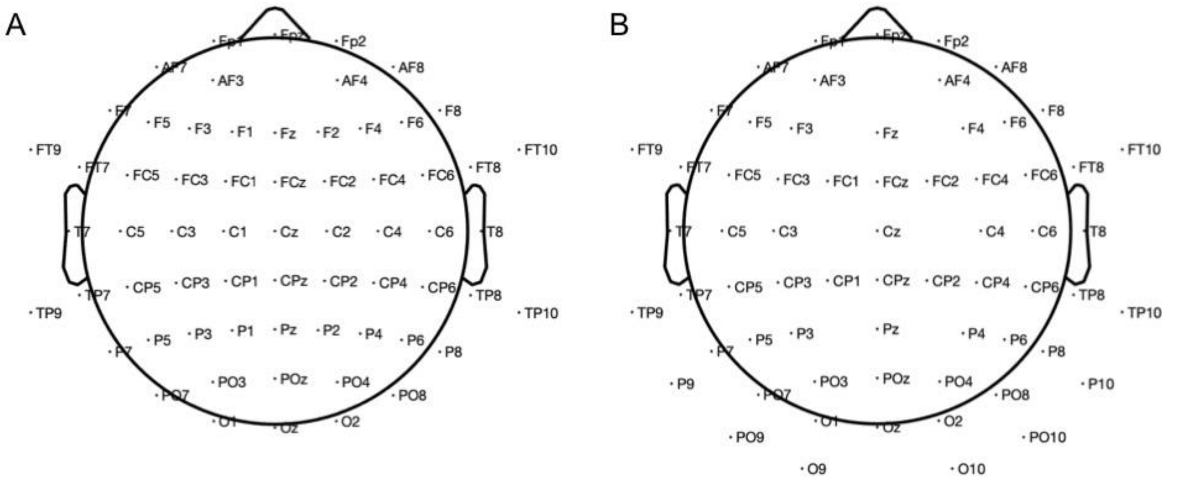

Figure S.9: Two different 64-channel EEG electrode layouts used for the resting-state dataset, when loaded in EEGLAB. 11 subjects were recorded using the layout shown in panel A whereas 5 subjects were recorded using the layout shown in panel B. Only subjects recorded with layout **A** were used for our analysis.

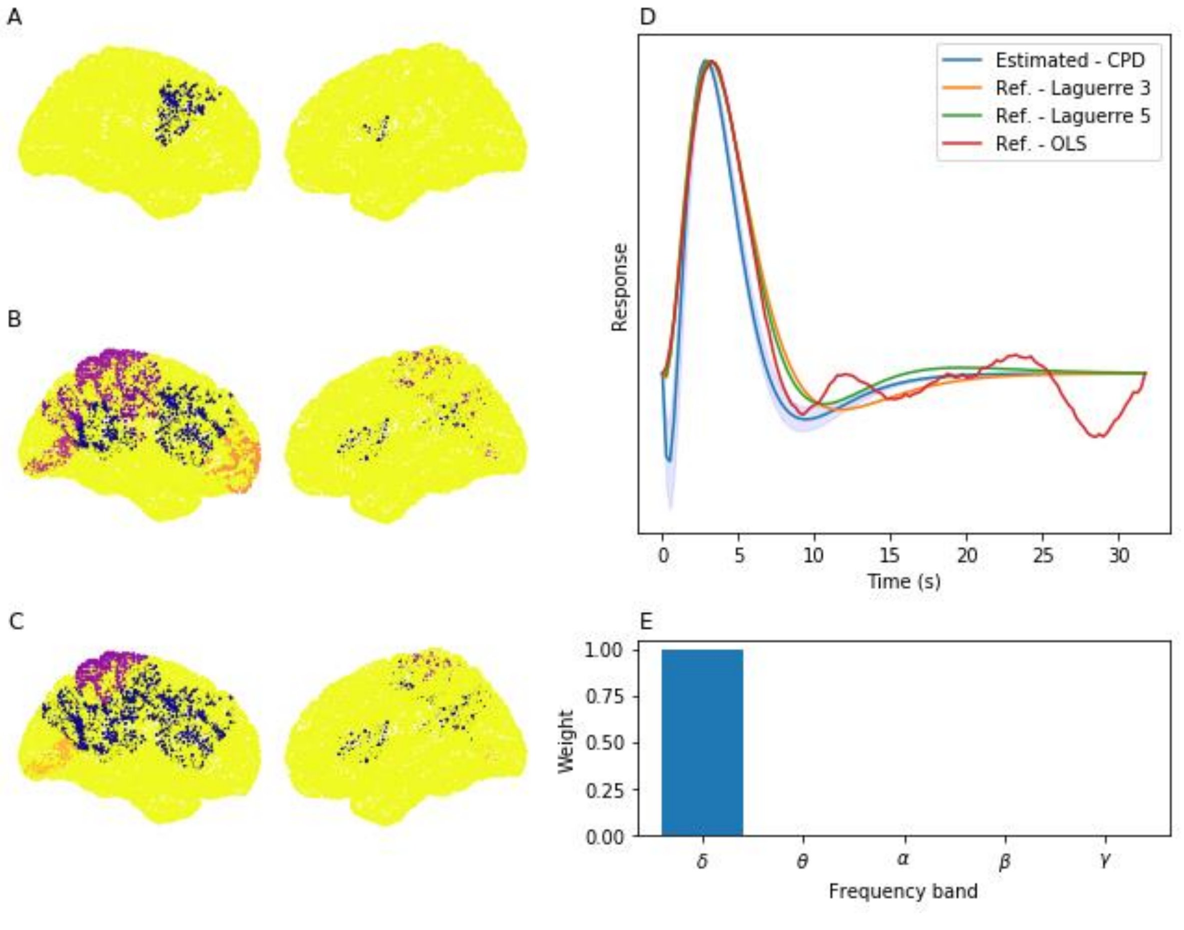

Figure S.10: Results for simulated data in right caudal anterior-cingulate cortex. A) Location of node of interest, results for which are shown in other subfigures. B) Estimated spatial distribution. C) Proxy of ground-truth for spatial distribution (i.e. reference distribution) obtained by correlating signals of slow subpopulations across nodes. D) Estimated and reference HRFs. Refer to SM 3 for methodology related to reference HRFs. Refer to Figure S.10D.2 for a comparison between estimated and reference HRFs where the post-stimulus undershoot is absent. E) Spectral distribution. Confidence intervals for panels D and E obtained using methodology described in SM 4. HRF: Hemodynamic Response Function, CPD: Canonical Polyadic Decomposition, OLS: Ordinary Least Squares.

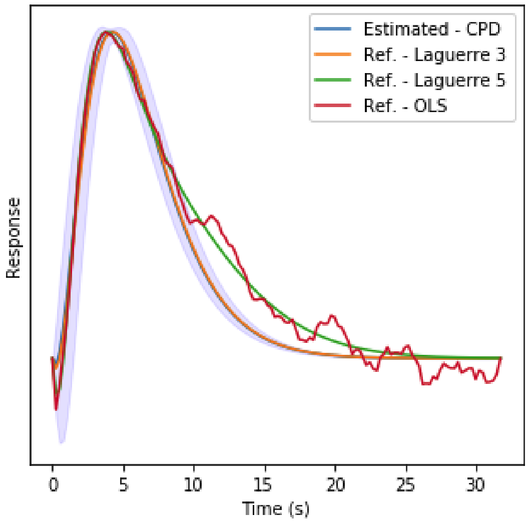

Figure S.10D.2: Modified replicate of Fig. S.10D, where the autoregulation parameter of the balloon model has been increased during simulation. This increase in parameter value results in the absence of a post-stimulus undershoot, as can be observed in this figure. Comparison between estimated HRF with CPD using 3 Laguerre functions and reference HRFs. HRF: Hemodynamic Response Function, CPD: Canonical Polyadic Decomposition, OLS: Ordinary Least Squares.

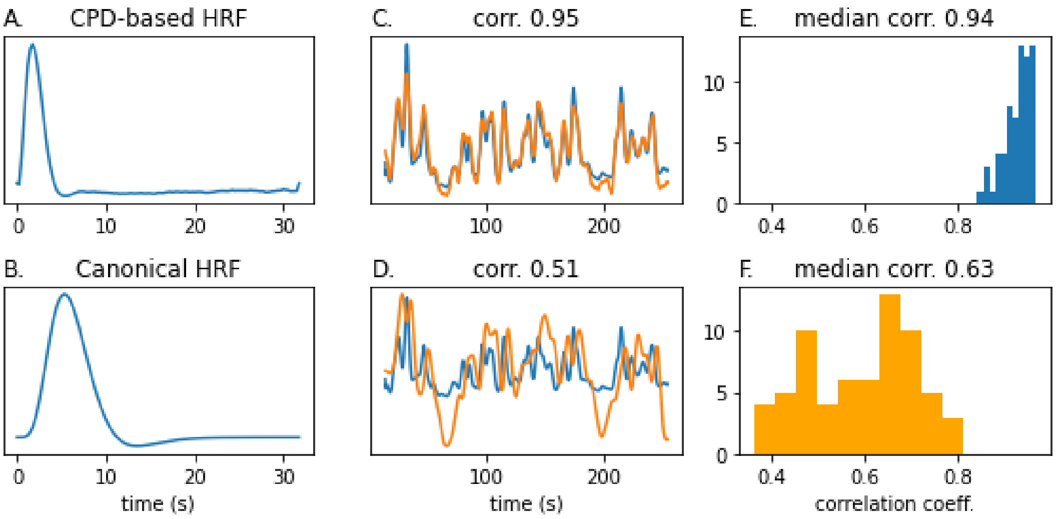

Figure S.11: Comparing the modelling capacity of a CPD-based HRF with that of the canonical HRF. BOLD signals were simulated for all 66 nodes of the cortical parcellation and used to extract spatial and spectral features as well as HRF estimated using our proposed CPD-based method. The simulated BOLD signals were then either estimated using all CPD outputs or by replacing the CPD-based HRF by the canonical HRF. For the BOLD signal simulations, the balloon model parameters were selected such that the resulting CPD-based HRF would differ from the canonical HRF to some extent. Specifically, the signal decay parameter of the balloon model was reduced from 1.54 seconds to 0.54 seconds. A) HRF derived using the CPD decomposition for randomly selected cortical region. B) Canonical HRF. C) Simulated BOLD signal (blue) overlaid with BOLD signal estimate (orange) when using CPD-based HRF for same cortical region as in A). D) Simulated BOLD signal (blue) overlaid with BOLD signal estimate (orange) when using canonical HRF for same cortical region as in A). E) Histogram of correlations when using CPD-based HRF for all 66 nodes of the cortical parcellation. F) Histogram of correlations when using canonical HRF for all 66 nodes of the cortical parcellation. corr: correlation.

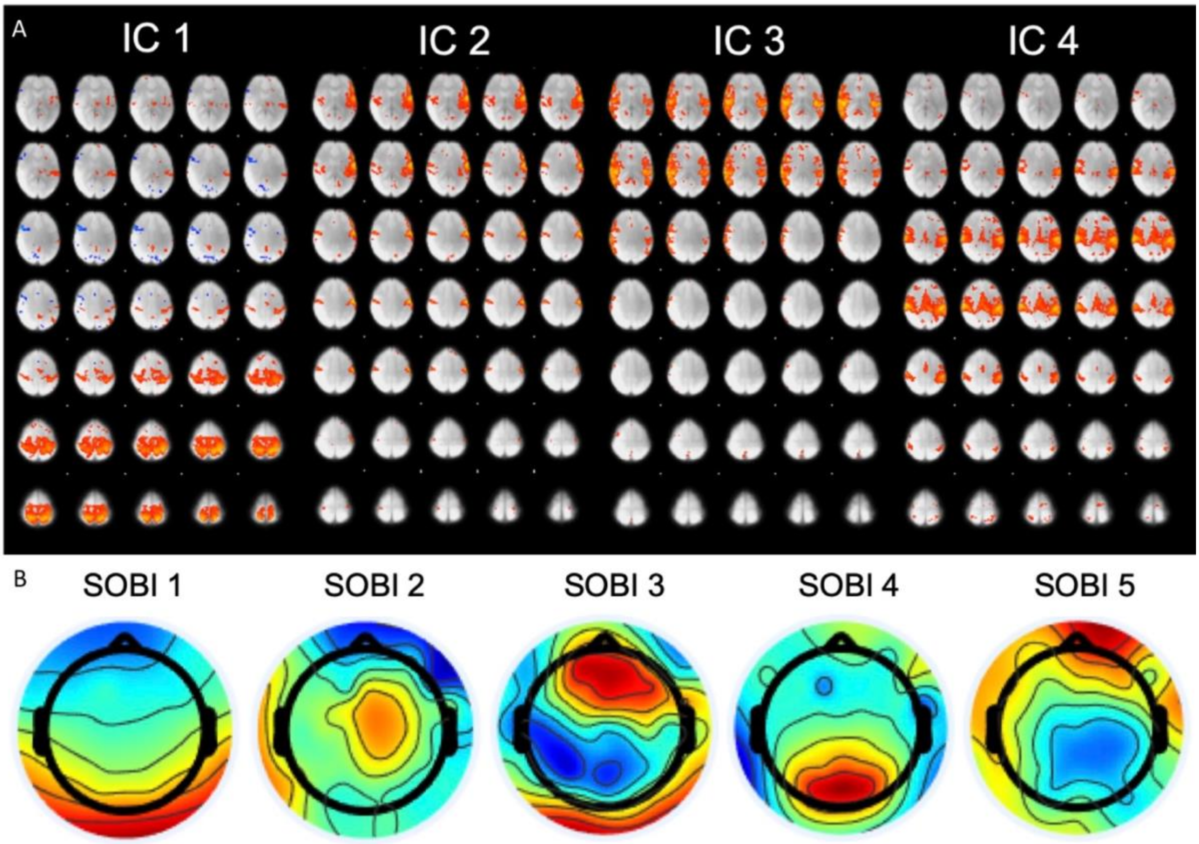

Figure S.12: BOLD independent components (IC) in panel A and scalp topography of EEG SOBI components in panel B for supplementary motor-imagery data. Main spatial coverage for BOLD IC1 is bilateral primary motor, primary somatosensory & premotor; for IC2 is left primary & secondary somatosensory cortex; for IC3 is right secondary somatosensory cortex & inferior parietal lobule; for IC4 is bilateral inferior & superior parietal lobules. Regions of BOLD ICs determined using Juëlich histological atlas.

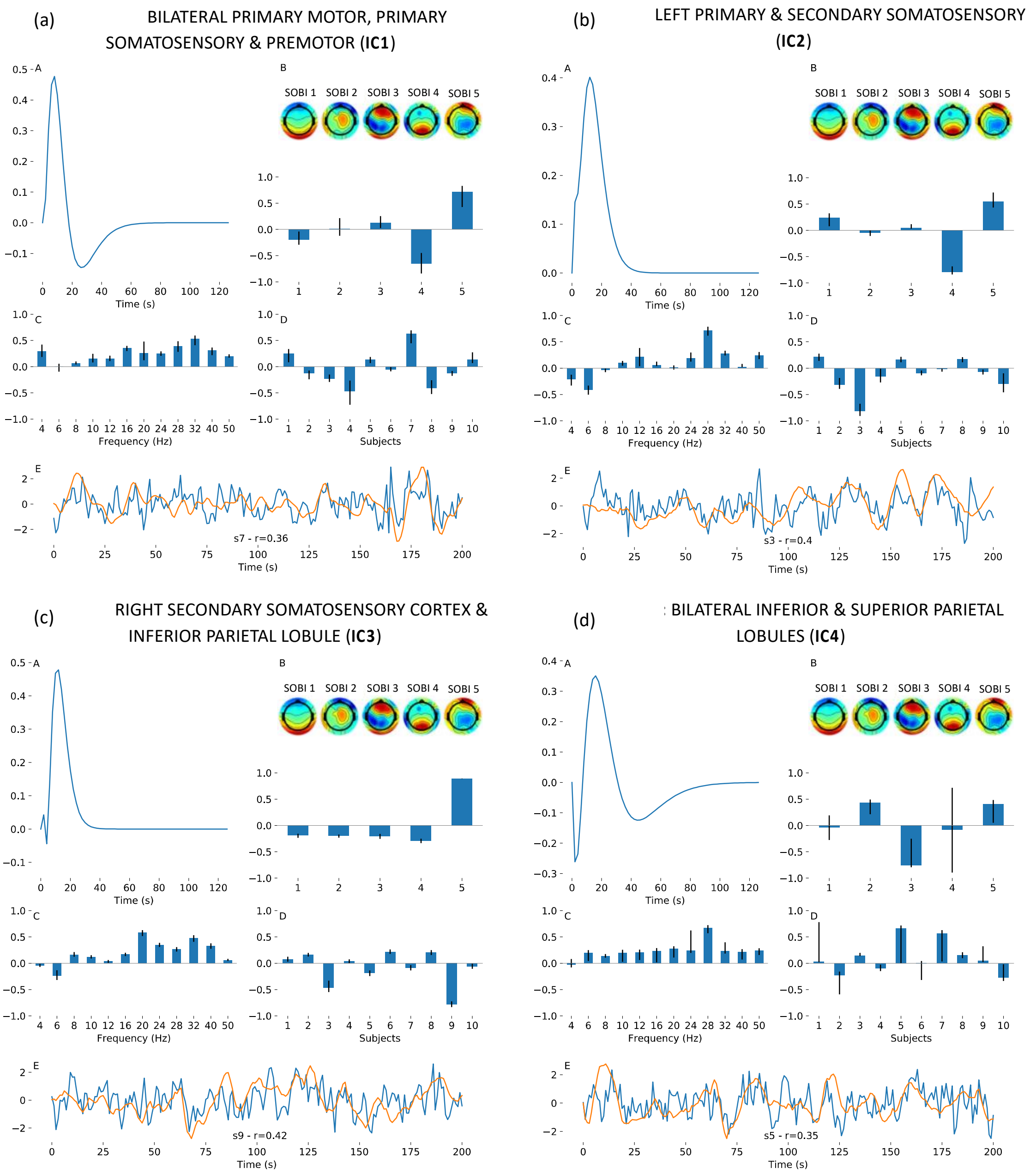

Figure S.13: Results for supplementary motor-imagery data when using 1 CPD component. Figure a-d shows results for different BOLD IC (Fig. S.12A). For each IC, estimated HRF shown in panel A, EEG SOBI topographies (Fig. S.12B) and spatial distribution in panel B, frequency distribution in panel C, subject distribution in panel D and BOLD signal estimate (orange) alongside real BOLD signal (blue) in panel E. For panel E, results presented for subject with highest correlation coefficient (r) between BOLD signal estimate and real BOLD signal. The vertical axis of each subfigure bears arbitrary units.

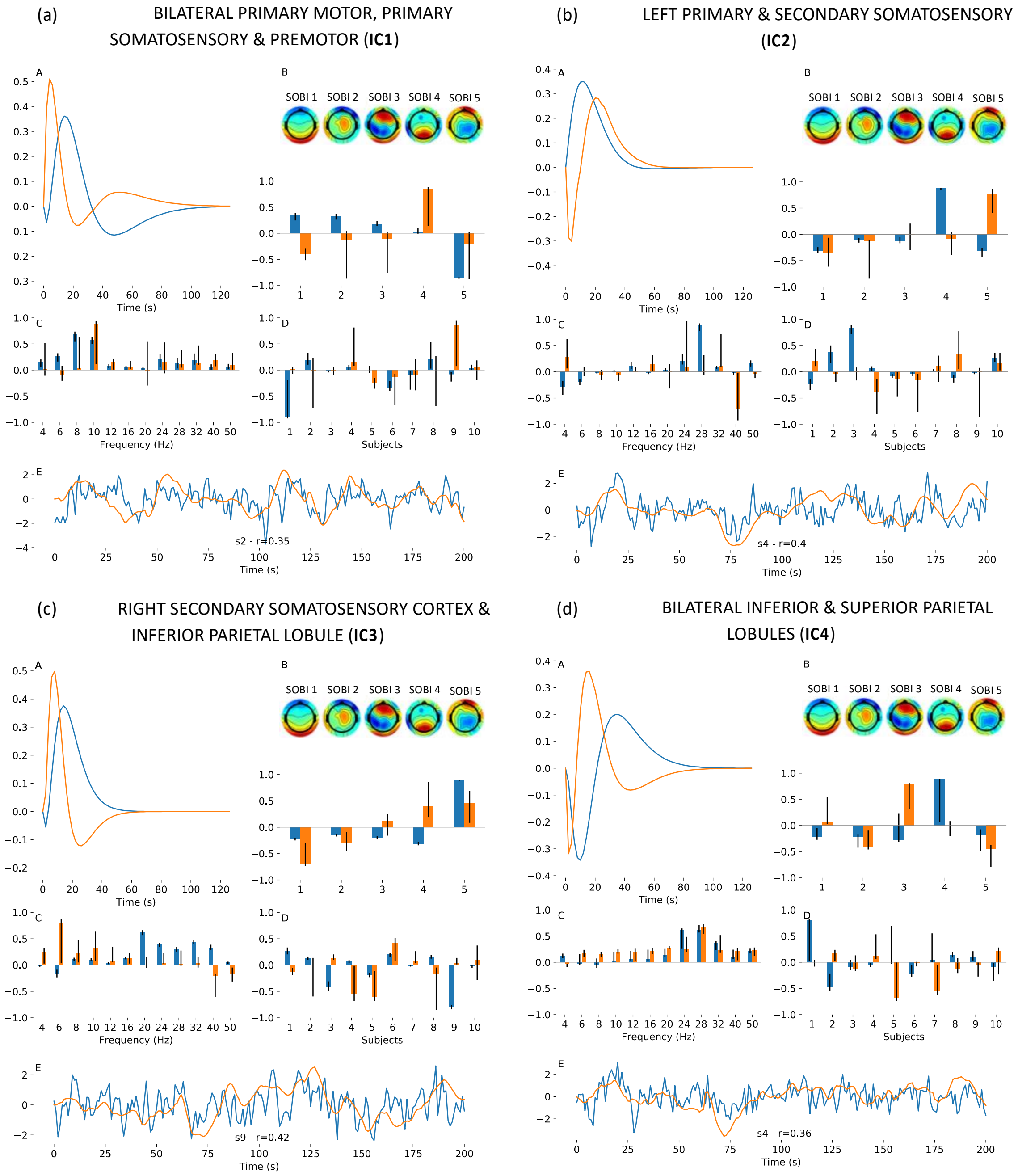

Figure S.14: Results for supplementary motor-imagery data when using 2 CPD components. Figure a-d shows results for different BOLD IC (Fig. S.12A). For each IC, estimated HRF shown in panel A, EEG SOBI topographies (Fig. S.12B) and spatial distribution in panel B, frequency distribution in panel C, subject distribution in panel D and BOLD signal estimate (orange) alongside real BOLD signal (blue) in panel E. For panel E, results presented for subject with highest correlation coefficient (r) between BOLD signal estimate and real BOLD signal. The vertical axis of each subfigure bears arbitrary units.

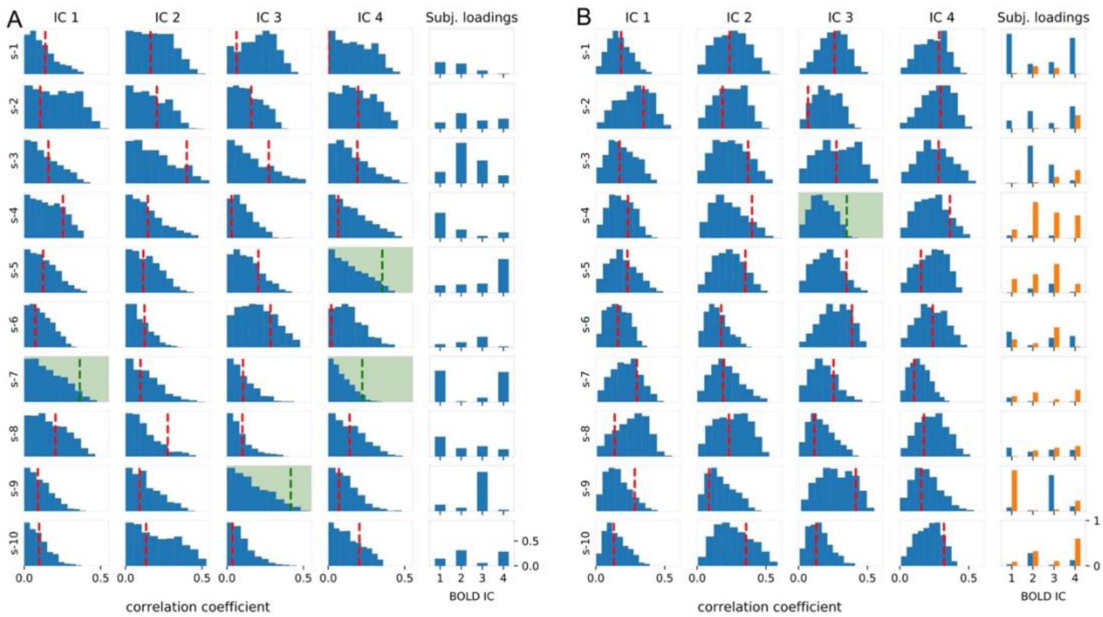

Figure S.15: Statistical inference of goodness-of-fit based on correlation coefficients for supplementary motor-imagery data. Panel A for 1 CPD component, panel B for 2 CPD components. For each BOLD IC and subject, correlation coefficient of BOLD estimate (vertical dotted line) is compared to null distribution (histogram). Dotted line is green if correlation coefficient exceeds 95th percentile of null distribution, corresponding to a *p*=0.05 statistical threshold, and dotted line is red if otherwise. Background is green when null hypothesis rejected. Far-right column of each panel shows absolute values of weightings for given subject across ICs. Main spatial coverage for BOLD IC1 is bilateral primary motor, primary somatosensory & premotor cortex; for IC2 is left primary & secondary somatosensory cortex; for IC3 is right secondary somatosensory cortex & inferior parietal lobule; for IC4 is bilateral inferior & superior parietal lobules.

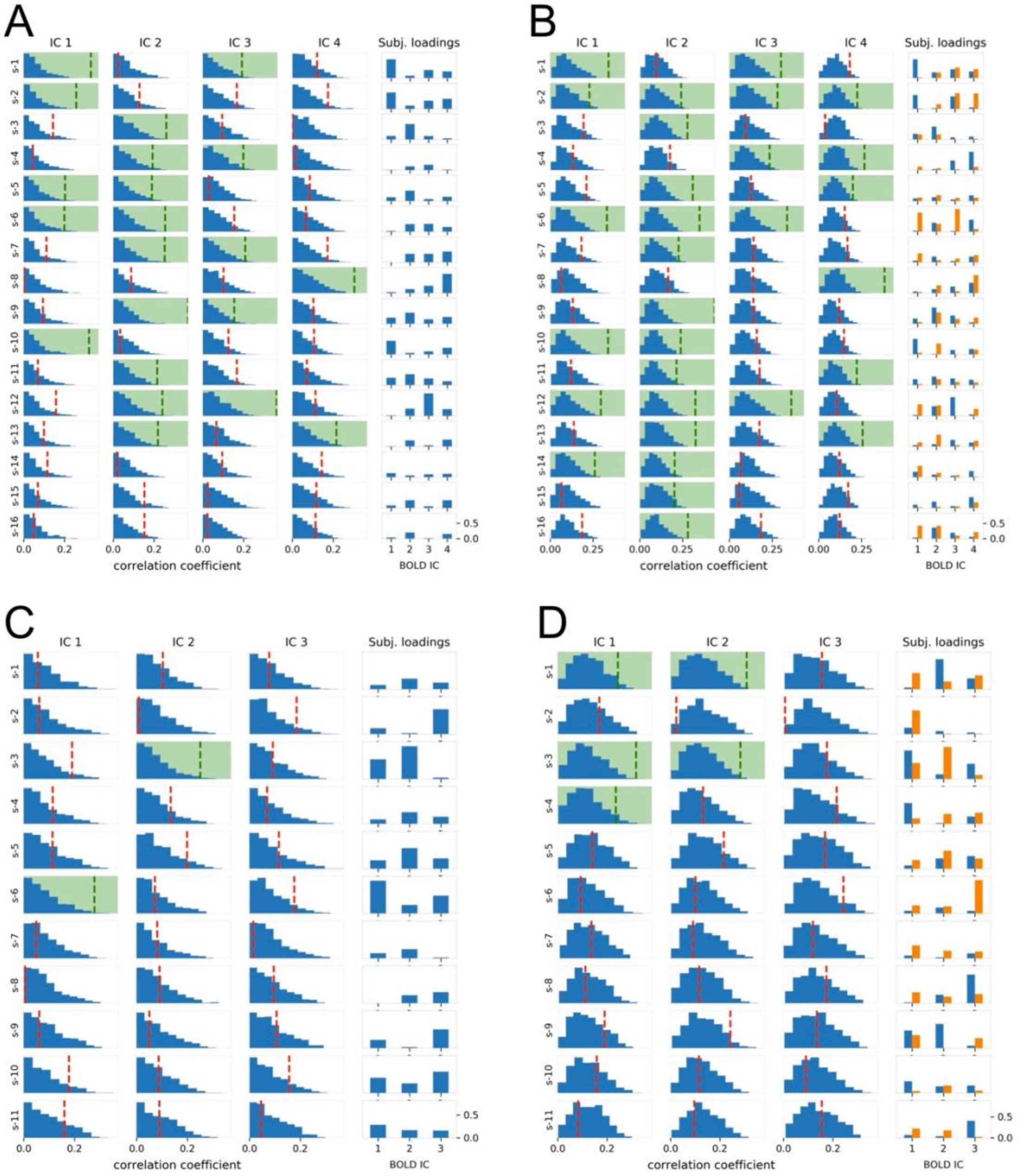

Figure S.16: Statistical inference of goodness-of-fit based on correlation coefficients when replacing phase-randomisation by bootstrapping. Motor-imagery data: panel A for 1 CPD component, panel B for 2 CPD components. Resting-state data: panel C for 1 CPD component, panel D for 2 CPD components. For each bold IC and subject, correlation coefficient of BOLD estimate (vertical dotted line) is compared to the null distribution (histogram). Dotted line is green if correlation coefficient exceeded the 95th percentile of null distribution, corresponding to a p=0.05 statistical threshold, and red if otherwise. Background is green when the null hypothesis was rejected. The far-right column of each panel shows absolute values of weightings across subjects and BOLD independent components (ICs). Spatial coverage for motor-imagery data (i.e. Panels A-B): IC1 – left primary motor & somatosensory cortex; IC2 – bilateral primary somatosensory cortex & inferior parietal lobule; IC3 – bilateral primary motor and somatosensory cortex; IC4 – bilateral premotor cortex. Resting-state networks (i.e. Panels C-D): IC1 – default mode network; IC2 – somatosensory network; IC3 – visual network.

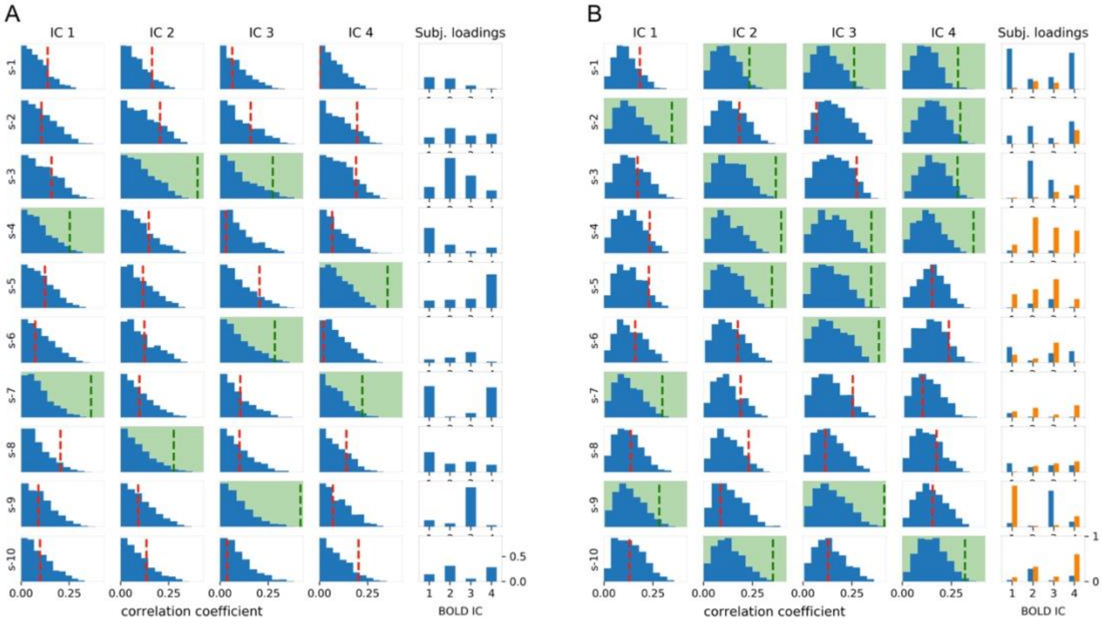

Figure S.17: Statistical inference of goodness-of-fit based on correlation coefficients when replacing phase-randomisation by bootstrapping. Results shown for supplementary motor-imagery data. Panel A for 1 CPD component, panel B for 2 CPD components. For each BOLD IC and subject, correlation coefficient of BOLD estimate (vertical dotted line) is compared to null distribution (histogram). Dotted line is green if correlation coefficient exceeds 95th percentile of null distribution, corresponding to a p=0.05 statistical threshold, and dotted line is red if otherwise. Background is green when null hypothesis rejected. Far-right column of each panel shows absolute values of weightings for given subject across ICs. Main spatial coverage for BOLD IC1 is bilateral primary motor, primary somatosensory & premotor cortex; for IC2 is left primary & secondary somatosensory cortex; for IC3 is right secondary somatosensory cortex & inferior parietal lobule; for IC4 is bilateral inferior & superior parietal lobules.
