# Supplementary material for: Extracting electrophysiological correlates of functional magnetic resonance imaging data using the canonical polyadic decomposition
Source: Hum Brain Mapp. 2022 May 14;43(13):4045–73. doi: 10.1002/hbm.25902 (PMC9374895; doi:10.1002/hbm.25902)
Supplement: Supplementary file 2 — TABLE S1 Decay‐rate parameter and percentile selected by the parameter sweep for each independent component (IC) of supplementary motor‐imagery dataset. Values shown when number of canonical polyadic decomposition (CPD) components set to 1 and 2. [file HBM-43-4045-s001.docx]

**TABLES (Supplementary Material):**

| Independent Component (IC) | decay-rate parameter | percentile |
| --- | --- | --- |
| 1 CPD component |  |  |
| IC1 | 2.6 | 0 |
| IC2 | 1.8 | 0 |
| IC3 | 1.4 | 25 |
| IC4 | 4.2 | 0 |
| 2 CPD components |  |  |
| IC1 | 4.6 | 50 |
| IC2 | 4.2 | 50 |
| IC3 | 2.4 | 25 |
| IC4 | 3.8 | 0 |

Table S.1: Decay-rate parameter and percentile selected by the parameter sweep for each independent component (IC) of supplementary motor-imagery dataset. Values shown when number of canonical polyadic decomposition (CPD) components set to 1 and 2.
